# Hypokalemic Hypochloremic Metabolic Alkalosis in Cystic Fibrosis: A Retrospective Observational Study with Clinical Characterization, *CFTR* Genetic Profiling, and a Comparative Genetic Analysis with Patients from a Brazilian Reference Center

**DOI:** 10.3390/ijms27177668

**Published:** 2026-08-27

**Authors:** Andressa Oliveira Peixoto, Larissa Ferreira Selicani, Camila Bento Safi, Daniela Souza Paiva Borgli, Adyléia Aparecida Dalbo Contrera Toro, Nathália Mariana Santos Sansone, Andrea de Melo Alexandre Fraga, Fernando Augusto Lima Marson, José Dirceu Ribeiro

**Affiliations:** 1 Pediatric Research Center, Faculty of Medical Sciences, University of Campinas (Unicamp—Universidade de Campinas), Campinas 13083-887, SP, Brazil; daniborgli@gmail.com (D.S.P.B.); leiadalbotoro@gmail.com (A.A.D.C.T.); andreafrag@gmail.com (A.d.M.A.F.); jdirceuribeiro@gmail.com (J.D.R.); 2 Faculty of Medical Sciences, University of Sapucaí Valley (Univás—Universidade do Vale do Sapucaí), Pouso Alegre 37550-000, MG, Brazil; larissaselicani@hotmail.com; 3 Faculty of Medical Sciences, Pontifical Catholic University of Campinas (PUC-Campinas—Pontifícia Universidade Católica de Campinas), Campinas 13086-900, SP, Brazil; cmlsafi23@gmail.com; 4 Laboratory of Molecular Biology and Genetics, Postgraduate Program in Health Sciences and Postgraduate Program in Health Data Science, São Francisco University (USF—Universidade São Francisco), Bragança Paulista 12916-900, SP, Brazil; nathaliasansone@hotmail.com; 5 Laboratory of Clinical and Molecular Microbiology, Postgraduate Program in Health Sciences and Postgraduate Program in Health Data Science, São Francisco University (USF—Universidade São Francisco), Bragança Paulista 12916-900, SP, Brazil; 6 LunGuardian Research Group—Epidemiology of Respiratory and Infectious Diseases, Postgraduate Program in Health Sciences and Postgraduate Program in Health Data Science, São Francisco University (USF—Universidade São Francisco), Bragança Paulista 12916-900, SP, Brazil

**Keywords:** *CFTR* genotype, cystic fibrosis, electrolytes, hypokalemia, metabolic alkalosis, Pseudo-Bartter syndrome

## Abstract

Cystic fibrosis is a genetic disorder caused by pathogenic variants in the Cystic Fibrosis Transmembrane Conductance Regulator (*CFTR*) gene and may be complicated by hypokalemic hypochloremic metabolic alkalosis (HHMA), resulting from excessive electrolyte loss through sweat. This retrospective study aimed to clinically characterize individuals with cystic fibrosis who developed HHMA, characterize their *CFTR* genetic profile and compare it with that of other individuals with cystic fibrosis followed at a Brazilian tertiary referral center, and contextualize these findings within the available national and international literature. Among 257 individuals with cystic fibrosis followed at a tertiary referral center between 2014 and 2024, 41 (15.9%) developed HHMA. Most patients were male (29; 70.7%), the median age at the first episode was approximately four months, and episodes occurred predominantly during summer. The biochemical profile was characterized by metabolic alkalosis, hyponatremia, hypokalemia, and hypochloremia. The F508del variant was the most common allele [F508del homozygous, 9 (22.0%); F508del heterozygous, 21 (51.2%); without the F508del variant, 11 (26.8%)], particularly among patients with recurrent episodes, which occurred in 9/11 (81.8%) cases. The most frequent clinical manifestations were dehydration, cough, and vomiting, although some patients presented with minimal symptoms. Management primarily consisted of intravenous and oral hydration, electrolyte replacement, and antibiotic therapy when indicated, with no HHMA-related deaths observed. The characterization of the *CFTR* genetic profile and its comparison with the broader cystic fibrosis population followed at the reference center provide additional insights into the genetic background of HHMA in a genetically diverse population. Taken together with findings from the national and international literature, these results indicate that HHMA is a relatively frequent and potentially recurrent complication of cystic fibrosis, particularly during early life, and may be influenced by both genetic and environmental factors. Early recognition, systematic electrolyte monitoring, and appropriate salt and fluid replacement may prevent severe complications and improve clinical management.

## 1. Introduction

Cystic fibrosis (CF, OMIM No. 219700) is an autosomal recessive genetic disease caused by pathogenic variants in the Cystic Fibrosis Transmembrane Conductance Regulator (*CFTR*) gene, which clinically manifests with a multisystemic phenotypic expression [1,2,3,4,5]. In addition to the well-known pulmonary and pancreatic morbidities, CF may present with acid–base disturbances and electrolyte imbalances, such as hypokalemic hypochloremic metabolic alkalosis (HHMA), a condition that mimics Bartter syndrome, a hereditary tubulopathy affecting the ascending limb of the loop of Henle [6,7].

Dysfunctional CFTR protein in the sweat glands leads to excessive loss of chloride and sodium ions through sweat, a loss that is intensified on hot days due to increased sweat production, resulting in significant sodium depletion, reduced extracellular fluid volume, and secondary activation of the renin–angiotensin–aldosterone system [8]. Elevated aldosterone levels promote compensatory sodium reabsorption and the secretion of potassium and hydrogen ions in the renal collecting ducts, leading to hypokalemia and metabolic alkalosis [9]. Regarding chloride depletion through perspiration, this loss results in increased bicarbonate reabsorption in the renal tubules, shifting potassium ions into the intracellular compartment, which further contributes to hypokalemia [9].

This condition is an uncommon but well-recognized complication of CF [10]. It occurs most frequently during the neonatal period and is uncommon in older children [10,11]. As the sole manifestation of CF, however, it is extremely rare [10]. Chronic fluid and electrolyte loss through sweat may be exacerbated by acute conditions such as gastrointestinal losses, respiratory infections, excessive perspiration in hot climates, and inadequate salt intake in infants [8]. Due to hyponatremia and hypokalemia, affected patients often have reduced appetite, further aggravating electrolyte imbalance [12].

Early diagnosis is essential due to potentially severe consequences. Metabolic alkalosis reduces ionized calcium levels, which may lead to muscle spasm, tetany, and seizures. In severe cases, it may reduce cardiac output, increase peripheral vascular resistance, and cause arrhythmias [13]. Additionally, it may reduce respiratory drive, predisposing to hypoventilation and respiratory complications, while increased mucus viscosity may favor infections, reinforcing the importance of electrolyte monitoring, particularly in warmer seasons [14].

We aimed to characterize the epidemiological and clinical features of HHMA in individuals with CF followed at a tertiary referral center, with emphasis on its frequency, biochemical profile, seasonality, recurrence, clinical presentation, and management, and to characterize the *CFTR* genetic profile of affected individuals and compare it with that of the broader CF population followed at the same center.

## 2. Results

### 2.1. Prevalence and Demographics

**Figure 1:**
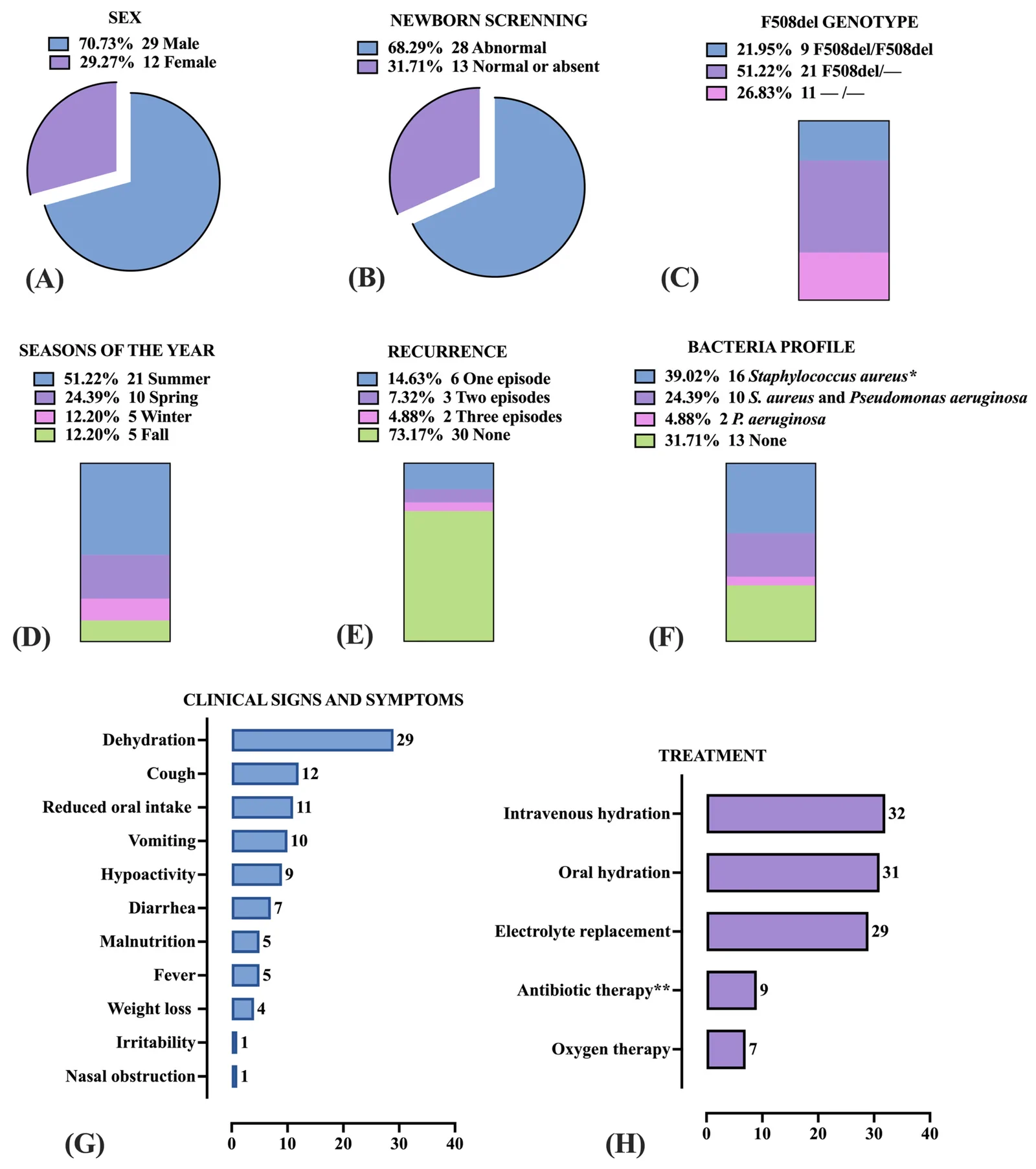
Genetic, demographic, clinical, microbiological, and treatment characteristics of patients with cystic fibrosis and hypokalemic hypochloremic metabolic alkalosis (HHMA). (**A**) sex distribution, (**B**) newborn screening results, (**C**) Cystic Fibrosis Transmembrane Conductance Regulator (*CFTR*) F508del genotype, (**D**) seasonal distribution of HHMA episodes, (**E**) recurrence profile, (**F**) bacterial profile, (**G**) clinical signs and symptoms, and (**H**) treatment strategies. Data are presented as number of patients and percentage [n (%)] or number of patients only, as appropriate. Other *CFTR* variants identified in the cohort are detailed in Table 1. For the seasonal distribution, recurrent HHMA episodes were identified, including 6 patients with one additional episode, 3 with two additional episodes, and 2 with three additional episodes. Recurrent episodes were analyzed separately in the seasonal distribution. *, One patient had co-detection of *S. aureus*, *Escherichia coli*, and *Moraxella catarrhalis*. **, Antibiotic therapy was administered both for decolonization and treatment of acute respiratory infections. Percentages may not sum exactly to 100% due to rounding. The symbol “—” indicates that the second allele is different from F508del and may correspond to another *CFTR* variant or, in rare cases, to no identifiable variant.

Among 257 individuals with CF followed at a tertiary referral center between January/2014 and January/2024, 41 (15.9%) patients fulfilled diagnostic criteria for HHMA. Of these patients, 29 (70.7%) were male, indicating a clear male predominance (Figure 1A, Supplementary Table S1). Age at the first HHMA episode ranged from 26 days to 13 years (Supplementary Table S1), with a median age of ~4 months, demonstrating that HHMA primarily manifests early in life but may also occur beyond infancy. This age distribution reinforces the vulnerability of infants and young children to hydroelectrolytic disturbances related to CFTR dysfunction.

Newborn screening was abnormal in 28 (68.3%) patients, normal in 6 (14.6%), and unavailable in the remaining cases (Figure 1B, Supplementary Table S1). Among patients with documented neonatal screening, all had undergone screening before the occurrence of HHMA. For the remaining participants, the available data were insufficient to establish whether HHMA preceded CF diagnosis. All patients had abnormal sweat test results, confirming the diagnosis of CF according to established diagnostic criteria (Supplementary Table S1) [1].

### 2.2. Biochemical Profile

**Figure 2:**
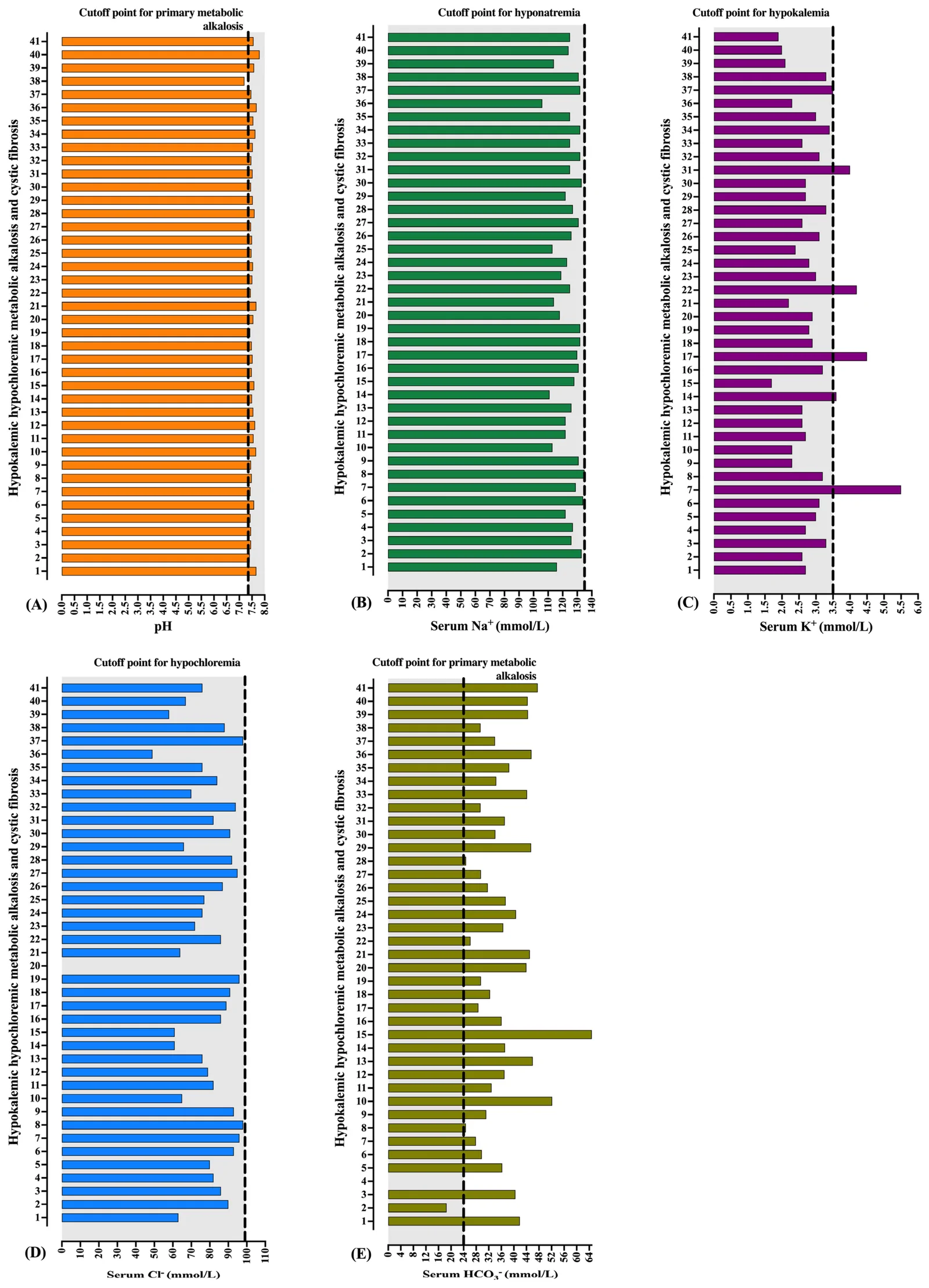
Distribution of arterial hydrogen potential (pH) (**A**), serum sodium (Na^+^; (**B**)), serum potassium (K^+^; (**C**)), serum chloride (Cl^−^; (**D**)), and serum bicarbonate (HCO_3_^−^; (**E**)) in patients with cystic fibrosis and hypokalemic hypochloremic metabolic alkalosis. Dashed lines indicate cutoff values for metabolic alkalosis (pH ≥ 7.45 and bicarbonate ≥ 24 mmol), hyponatremia (Na^+^ ≤ 135 mmol/L), hypochloremia (Cl^−^ ≤ 99 mmol), and hypokalemia (K^+^ ≤ 3.5 mmol). mmol/L: millimoles per liter.

Laboratory findings obtained before treatment were consistent with the classical biochemical pattern of HHMA, showing median plasma values of potential of hydrogen (pH) 7.52 (IQR, 7.48–7.58), sodium of 126 (IQR, 122–131) mmol/L, potassium of 2.7 mmol/L, chloride of 82 (IQR, 71.5–90.3) mmol/L, and bicarbonate of 36.5 (IQR, 29.7–44.1), as detailed in Figure 2 and Supplementary Table S2. These findings are consistent with HHMA secondary to excessive salt loss.

### 2.3. Genotype and Seasonality

Regarding *CFTR* genotypes, the most frequent category among patients with HHMA was F508del heterozygosity (21; 51.2%), followed by F508del homozygosity (9; 22.0%) and genotypes without the F508del variant (11; 26.8%) (Figure 1C). The detailed genotypic and *CFTR* variant-class profiles of the 41 patients with HHMA are presented in Table 1, which illustrates the substantial genetic heterogeneity of this group, including variants from Classes I to V. To provide a comprehensive comparison with the non-HHMA group, the genotypic profile of the 216 patients without HHMA is presented in Table 2. This comparison further demonstrates the predominance of F508del-containing genotypes in both groups, while also highlighting the broad spectrum of *CFTR* variants and variant classes identified in the study population.

HHMA episodes occurred throughout the year but were more frequent during summer (21; 51.2%) (Figure 1D, Supplementary Table S1). This seasonal pattern strongly supports the role of environmental temperature, sweating, and insensible salt loss as triggering factors for HHMA in individuals with CF [10].

### 2.4. Recurrence

Recurrence of HHMA was documented in 11 (26.8%) patients, accounting for 18 recurrent episodes, highlighting that HHMA may represent a recurrent metabolic complication rather than a single isolated event (Supplementary Table S3). Six patients experienced one additional episode, three experienced two additional episodes, and two experienced three additional episodes (Figure 1E, Supplementary Table S3). The age at recurrence ranged from 2 months and 11 days to 3 years and 9 months. Among the 18 recurrent episodes, 11 (61.1%) occurred during summer, five (27.8%) during spring, and two (11.1%) during fall, with no recurrent episodes reported during winter (Supplementary Table S3).

Among patients with recurrence, the majority carried at least one F508del allele, consistent with the association between F508del-containing genotypes and recurrent salt-loss phenotypes [3,13]. The predominance of both HHMA episodes overall and recurrent episodes during the warmer months supports the potential contribution of environmental temperature, increased sweating, and insensible salt and fluid losses as triggering factors for HHMA in individuals with CF [10].

### 2.5. Microbiological Findings, Clinical Presentation, and Management

Dehydration was the most frequent manifestation (29; 70.7%), followed by cough (12; 9.3%) and reduced oral intake (11; 26.8%), as summarized in Figure 1G and Supplementary Table S1. Vomiting, hypoactivity, and failure to thrive were also documented, particularly among infants. Notably, three patients (7.3%) were oligosymptomatic at the time of HHMA diagnosis, emphasizing that HHMA may be detected primarily through laboratory abnormalities and underscoring the importance of routine electrolyte monitoring in patients with CF.

Airway microbiology findings were available for patients with documented cultures or previous colonization. *Staphylococcus aureus* was the most frequently identified microorganism, either alone or in combination with *Pseudomonas aeruginosa* (Figure 1F, Supplementary Table S2). Although microbiological findings were not a primary outcome of this study, they provide clinical context regarding concurrent or previous respiratory colonization among patients experiencing HHMA.

Treatment strategies primarily included intravenous hydration (32; 78.0%), oral hydration (31; 75.6%), and electrolyte replacement (29; 70.7%) (Figure 1H and Supplementary Table S3). Antibiotic therapy was administered in 9 (21.9%) cases, mainly for acute respiratory infections or bacterial decolonization. Oxygen therapy was required in seven (17.1%) patients, including three who required invasive mechanical ventilation (Figure 1H and Supplementary Table S3). Additional individualized interventions included nutritional support via enteral or parenteral routes, bronchodilators, dornase alfa, and vitamin supplementation.

No HHMA-related deaths were identified, suggesting that timely recognition and appropriate management may prevent severe outcomes.

Individual patient-level clinical, biochemical, seasonal, recurrence, microbiological, and treatment characteristics are provided in Supplementary Tables S1–S3 to illustrate the clinical heterogeneity of HHMA presentations in this cohort. The data are presented in Figure 1 and Figure 2.

### 2.6. CFTR Genotype and Sex According to HHMA Status

Among the 257 patients included in the analysis, 41 (16.0%) had HHMA, whereas 216 (84.0%) did not. The distribution of the F508del genotype did not differ significantly between patients with and without HHMA. The homozygous F508del/F508del genotype was identified in 9 (22.0%) patients with HHMA and 68 (31.5%) patients without HHMA (OR = 0.614, 95% CI: 0.237–1.591; *p*-value = 0.340), while the F508del/— genotype was observed in 21 (51.2%) and 97 (44.9%) patients, respectively (OR = 1.004, 95% CI: 0.449–2.244; *p*-value = 1.000), using the —/— genotype as the reference (Table 3).

In contrast, a significant difference was observed when *CFTR* genotypes were grouped according to variant classes. Two *CFTR* variants from classes I, II, and/or III were present in 39 (95.1%) patients with HHMA compared with 160 (74.1%) patients without HHMA, corresponding to significantly higher odds of HHMA (OR = 6.83, 95% CI: 1.60–29.19; *p*-value = 0.002) (Table 3). Conversely, variants from classes III, IV, and/or VI, or unclassified/not identified variants, were more frequent among patients without HHMA.

Sex was also significantly associated with HHMA. Male patients accounted for 29 (70.7%) of those with HHMA compared with 106 (49.1%) of those without HHMA, resulting in higher odds of HHMA among males (OR = 2.51, 95% CI: 1.22–5.17; *p*-value = 0.016) (Table 3). Female patients represented 12 (29.3%) of the HHMA group and 110 (50.9%) of the non-HHMA group.

### 2.7. Narrative Review

**Figure 3:**
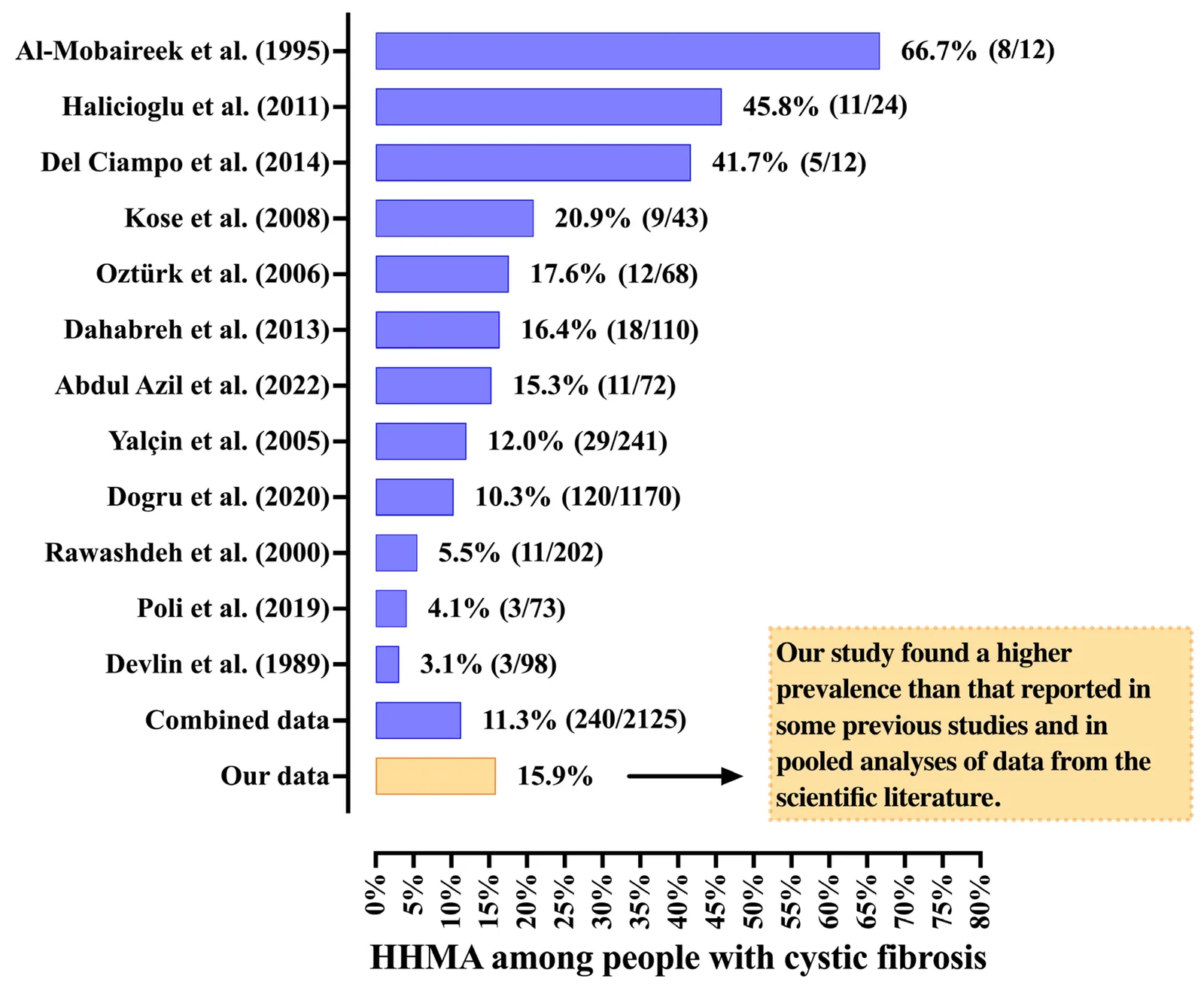
Prevalence of hypokalemic hypochloremic metabolic alkalosis (HHMA) among patients with cystic fibrosis (CF) reported in different studies. The bar chart compares the prevalence of HHMA in patients with CF found in our study (15.9%, 41/257 cases) with that reported by Devlin et al. (1989), Poli et al., (2019), Rawashdeh et al. (2000), Dogru et al. (2020), Yalçin et al. (2005), Abdul Azil et al. (2022), Dahabreh et al. (2013), Oztürk et al. (2006), Kose et al. (2008), Del Ciampo et al. (2014), Al-Mobaireek et al. (1995), and Halicioglu et al. (2011) [11,13,29,30,31,32,33,34,37,59,60,61]. The aggregated prevalence across these studies was 11.3% (240/2125), based on the total number of HHMA cases and individuals reported in the included studies. The prevalence observed in our study was higher than that reported in several individual studies and than the aggregated prevalence across the included studies. These findings highlight the importance of clinical awareness of HHMA in patients with CF, particularly in regions with warm climates and limited access to neonatal screening. Data are presented as percentages (number of patients with HHMA/number of patients with CF evaluated).

To provide context for and facilitate the interpretation of the findings of the present study, which investigated HHMA in individuals with CF at a Brazilian referral center, a narrative literature review was conducted. The main characteristics, clinical and genetic findings, and conclusions of the included studies are summarized in Figure 3, Supplementary Tables S4 and S5 [7,8,9,10,11,12,13,14,29,30,31,32,33,34,35,36,37,38,39,40,41,42,43,44,45,46,47,48,49,50,51,52,53,54,55,56,57,58,59,60,61].

## 3. Discussion

In this retrospective study, HHMA was identified in 16.0% of individuals with CF, predominantly occurring in early life, with a recurrence rate of 26.8%, strong associations with severe *CFTR* genotypes and summer seasonality, and no related mortality.

The prevalence of HHMA observed in our cohort should be interpreted within the specific epidemiological, geographic, and healthcare context of Brazil. Previous Brazilian studies have reported substantial variability in the occurrence of HHMA among individuals with CF. In a cohort of infants diagnosed through newborn screening in Ribeirão Preto, São Paulo, HHMA was reported in 41.6% of 12 infants with CF [35]. Similarly, a more recent Brazilian case series from Bahia reported HHMA in 50% of 14 children with delayed CF diagnosis following false-negative or unsuccessful newborn screening [62]. In addition, a Brazilian case report described HHMA in a 5-year-old child with CF who developed severe electrolyte and acid–base disturbances during the summer in Brazil [63]. Although these studies differ substantially in design, age distribution, and clinical context, their findings indicate that HHMA is not an isolated manifestation in the Brazilian CF population and may represent a clinically relevant complication, particularly among infants and children.

The Brazilian literature is further supported by reports from other settings describing HHMA as an important early manifestation of CF. Fustik et al. reported HHMA in 18.8% of 85 infants newly diagnosed with CF, with a mean age of 3.5 months, highlighting the occurrence of this metabolic complication during infancy and its potential to represent the first manifestation of CF [64]. Similarly, Yalçin et al. identified HHMA in 12% of 241 children with CF, with a median age of 4 months at the first episode; recurrent episodes requiring hospitalization occurred in 12 of 29 affected patients [13]. More recently, Lopes et al. described HHMA as the initial presentation of CF in an infant hospitalized during the summer with dehydration, oliguria, and severe electrolyte and acid–base abnormalities, reinforcing the importance of recognizing this condition in young children, particularly during periods of increased sweating and additional sodium and chloride losses [6].

Environmental conditions, particularly high ambient temperatures, may contribute to the occurrence of HHMA by increasing sweat-mediated sodium and chloride losses in individuals with CF. The study conducted in Ribeirão Preto, a region characterized by a warm climate, specifically discussed climatic conditions as a potential contributing factor to the occurrence of HHMA [35]. The Brazilian case report by Lopes et al. also described the occurrence of severe HHMA during the summer, in association with gastrointestinal and urinary fluid losses, further illustrating how increased environmental temperature and additional losses may increase susceptibility to electrolyte disturbances [6]. However, these observations should be interpreted as a plausible association rather than evidence of a direct causal effect of climate. The occurrence of HHMA is likely influenced by multiple interacting factors, including age, nutritional status, pancreatic insufficiency, intercurrent gastrointestinal or respiratory illness, sodium intake, *CFTR* genotype, newborn screening performance, and access to specialized CF care.

Importantly, the Brazilian studies do not establish that climate directly causes a higher prevalence of HHMA. Rather, the findings from Ribeirão Preto and the Brazilian case report, together with the seasonal pattern observed in our cohort, support a biologically plausible association between warmer environmental conditions and increased risk of salt and fluid depletion. This interpretation is consistent with the pathophysiology of CF, in which defective CFTR-mediated chloride and sodium reabsorption in sweat ducts results in increased electrolyte losses, which may become clinically relevant when sweating is intensified. Therefore, environmental temperature should be considered a potential modifying factor rather than an independent causal determinant of HHMA.

Comparison with previously published Brazilian studies should therefore be interpreted cautiously because of differences in study design, sample size, age distribution, diagnostic strategies, clinical characteristics, and healthcare settings. The Ribeirão Preto study included infants identified through newborn screening [35], whereas the Bahia case series specifically evaluated children with delayed CF diagnosis after false-negative or unsuccessful newborn screening [62]. In contrast, the third Brazilian publication was a single case report involving a preschool child with severe electrolyte disturbances during the summer and concomitant gastrointestinal losses [63]. These differences may substantially influence both the observed prevalence and the clinical severity of HHMA. Therefore, the prevalence observed in our study should not be considered representative of the Brazilian CF population as a whole, particularly because our cohort was derived from a single specialized CF reference center. Nevertheless, the occurrence of HHMA in 41.6% of infants in the Ribeirão Preto cohort, 50% of children in the Bahia series, and the additional Brazilian case report demonstrates that this complication has been repeatedly documented in Brazil, despite the substantial heterogeneity of the available evidence. These findings, together with the 15.9% prevalence observed in our cohort, support the clinical relevance of HHMA in the Brazilian context and reinforce the importance of monitoring electrolyte disturbances in individuals with CF, particularly during early life and periods of high environmental temperature or increased fluid losses.

Importantly, the present study extends previous reports by providing a longitudinal characterization of HHMA in a relatively large cohort of individuals with CF followed at a single Brazilian referral center over a 10-year period. Beyond estimating the prevalence of HHMA, our study describes its age distribution, recurrence, seasonal pattern, clinical and biochemical characteristics, treatment, and, particularly, its genetic profile. The revised analysis includes a detailed comparison of *CFTR* genotypes between individuals with and without HHMA, including F508del status and the distribution of variants according to functional classes. This approach provides additional information on the genetic background of HHMA within the broader CF population and may help clarify potential genotype-related susceptibility to salt-wasting episodes. Nevertheless, the multifactorial nature of HHMA suggests that genetic background alone is unlikely to fully explain its occurrence. Host factors, epithelial ion transport, environmental exposure, nutritional status, and intercurrent infections or gastrointestinal losses may interact to determine the clinical expression of salt and fluid depletion.

Emerging evidence also suggests that epithelial responses to infection and inflammation may involve complex regulatory mechanisms that extend beyond CFTR dysfunction itself. Studies investigating host ribonucleic acid (RNA)-binding proteins have demonstrated their potential role in regulating infection-associated epithelial apoptosis and epithelial–mesenchymal changes [65]. Although these mechanisms were not directly evaluated in the present study and no causal relationship with HHMA can be established from our data, they provide a broader biological context in which host responses, epithelial integrity, and infection-related processes may potentially influence the susceptibility or clinical expression of electrolyte disturbances in CF. Future studies integrating *CFTR* genotype, epithelial function, inflammatory responses, infection status, and environmental exposure may help determine whether these pathways contribute to the heterogeneity of HHMA.

HHMA is a well-recognized electrolyte complication of CF and results from excessive sodium and chloride loss through sweat caused by CFTR dysfunction. In CF, impaired reabsorption of sodium and chloride in sweat ducts leads to extracellular volume depletion and activation of the renin–angiotensin–aldosterone system, resulting in renal potassium and hydrogen ion loss and hypokalemic metabolic alkalosis [8,10]. This mechanism directly explains the biochemical profile observed in our cohort. Infants are particularly vulnerable due to their higher body surface area–to–weight ratio, immature renal compensatory mechanisms, and potentially insufficient sodium intake. Severe *CFTR* variants, including F508del, may also be associated with more pronounced salt-loss phenotypes and recurrent metabolic disturbances [58,66]. Additionally, environmental factors such as high temperatures may increase sweat losses, while sex-related differences in sweat gland activity may contribute to the higher prevalence observed among males [11,13,67,68].

In this cohort, the prevalence of HHMA was higher than that reported in most international series [57]. This finding may reflect climatic conditions, increased clinical awareness, early diagnosis of CF through newborn screening, and systematic biochemical surveillance at a specialized referral center [13]. The predominance of the F508del variant aligns with global CF genetic epidemiology and supports its association with severe salt-loss phenotypes and recurrent metabolic disturbances [3]. The observed recurrence rate suggests that HHMA should be considered a long-term management issue rather than a transient neonatal phenomenon. This interpretation is consistent with the study by Yalçin et al., in which recurrent HHMA occurred in 12 of 29 affected children and was observed up to 48 months of age [13].

The clinical presentation of HHMA may range from mild electrolyte abnormalities to severe dehydration and neurological or cardiovascular complications. Yalçin et al. reported vomiting, loss of appetite, and dehydration as common manifestations among children with CF and HHMA [13], while Lopes et al. described an infant presenting with dehydration, oliguria, apathy, hypochloremic metabolic alkalosis, hypokalemia, and hyponatremia [6]. These observations emphasize that HHMA may occur even when classical pulmonary or gastrointestinal manifestations of CF are not prominent and that its recognition requires a high index of suspicion.

Sweat glands in individuals with CF, in contrast to those in healthy individuals, do not secrete sweat normally in response to β-adrenergic stimulation but retain responsiveness to cholinergic stimulation. Men generally exhibit a lower number of active sweat glands; however, they demonstrate a higher sweat secretion rate per gland compared with women, which may partly explain the higher prevalence observed among males in our study [69]. Nevertheless, sex differences should be interpreted cautiously because the mechanisms underlying HHMA are multifactorial and may be strongly influenced by age, genotype, environmental exposure, nutritional status, and intercurrent illness.

Early recognition of HHMA is essential, as metabolic alkalosis may lead to neuromuscular irritability, tetany, seizures, hypoventilation, cardiac arrhythmias, and worsening pulmonary disease due to increased mucus viscosity and impaired mucociliary clearance [10]. Moreover, HHMA may represent the first clinical manifestation of CF, particularly in infants, and should prompt diagnostic investigation even in the absence of classic respiratory or gastrointestinal symptoms [10,13]. This diagnostic relevance is supported by Fustik et al., who found HHMA in 18.8% of newly diagnosed infants and emphasized that CF should be considered in infants presenting with metabolic alkalosis and hypoelectrolytemia, regardless of the presence of respiratory or gastrointestinal manifestations [64]. Similarly, Lopes et al. emphasized that HHMA may be an initial manifestation of CF and that clinical awareness is essential for timely diagnosis [6].

From a clinical perspective, our findings reinforce the importance of systematic electrolyte monitoring in patients with CF, particularly in early life and during warmer seasons, together with proactive counseling on adequate salt and fluid intake. Preventive salt supplementation and hydration strategies should be reinforced during periods of increased sweating or intercurrent illness, particularly in patients with previous HHMA episodes. Furthermore, clinicians should recognize HHMA as a potential presenting feature of CF.

Preventive strategies should also be incorporated into the routine care of individuals with CF who are at risk of HHMA [70]. Adequate sodium and fluid intake is particularly important during hot weather, periods of increased sweating, fever, respiratory or gastrointestinal illness, and reduced oral intake, when salt and water losses may increase substantially. Caregivers should receive clear guidance regarding adequate salt supplementation according to age and clinical circumstances, as well as appropriate hydration and early recognition of warning signs such as reduced oral intake, vomiting, lethargy, weight loss, or dehydration. Patients with a previous episode of HHMA may benefit from closer electrolyte monitoring during high-risk periods, given the recurrence observed in our cohort [6,9,71]. These preventive measures may help reduce the risk of severe electrolyte disturbances and facilitate earlier intervention.

The treatment approaches described in previous studies also support the importance of early recognition and electrolyte replacement. In the cohort reported by Yalçin et al., all patients received intravenous fluids and sodium chloride supplementation, reflecting the severity of the episodes in that population [13]. In contrast, the Ribeirão Preto cohort demonstrated that early detection through systematic screening and oral electrolyte replacement was sufficient in all five affected infants, with relatively mild clinical manifestations [35]. The Brazilian case reported by Lopes et al. required intravenous rehydration and sodium and chloride supplementation, with complete recovery [6]. Taken together, these studies suggest that the clinical severity of HHMA may vary considerably and that early detection may allow less intensive treatment and potentially prevent severe complications.

An additional consideration is the progressive availability of CFTR-modulator therapies in Brazil during the study period. Access to these therapies was initially restricted to specific genetic and clinical indications. Ivacaftor was incorporated into the Brazilian Unified Health System (SUS—Sistema Único de Saúde) in 2021 for patients with specific responsive *CFTR* variants [72,73], whereas lumacaftor/ivacaftor and tezacaftor/ivacaftor were not incorporated into the SUS. Elexacaftor/tezacaftor/ivacaftor was subsequently incorporated into the SUS in September 2023 for eligible patients aged ≥ 6 years carrying at least one F508del variant [74,75].

Importantly, none of the 41 patients in our cohort was receiving CFTR-modulator therapy at the time of the HHMA episode. Thus, all HHMA episodes analyzed in the present study occurred without concomitant CFTR-modulator exposure. This context is relevant when interpreting our findings, as the cohort predominantly reflects the clinical characteristics of HHMA before the widespread availability of highly effective CFTR-modulator therapy in Brazil. Importantly, emerging evidence suggests that highly effective CFTR modulation may directly influence salt and fluid homeostasis. In a recent study of 50 individuals with CF initiating elexacaftor/tezacaftor/ivacaftor, treatment increased plasma sodium concentration and the diuretic response to sodium and volume loading, while reducing aldosterone levels, venous total CO_2_, and the proportion of patients with low plasma sodium, suggesting improved sodium chloride and fluid conservation [76]. These findings raise the possibility that highly effective CFTR modulation may reduce susceptibility to salt-wasting and HHMA. Therefore, the absence of CFTR-modulator exposure in our cohort is particularly relevant, as the prevalence and recurrence patterns observed may differ in contemporary populations with broader access to highly effective modulator therapy.

The potential effect of CFTR modulators on HHMA also highlights an important area for future research. If restoration of CFTR function improves epithelial sodium and chloride handling, as suggested by Berg et al. [76], widespread access to highly effective modulator therapy could alter the epidemiology, recurrence, and severity of salt-wasting episodes. However, these effects should not be extrapolated directly to infants or to all individuals with CF, particularly because access to modulators, age at initiation, genotype, and treatment eligibility vary substantially. Prospective studies evaluating electrolyte balance, sweat electrolyte concentrations, sodium requirements, and HHMA incidence before and after CFTR-modulator initiation will be important to determine whether the apparent correction of salt-wasting translates into a clinically meaningful reduction in HHMA.

Overall, our findings, considered together with previous Brazilian and international evidence, indicate that HHMA remains a clinically relevant complication of CF, particularly during early life and under conditions that increase fluid and electrolyte losses. The Brazilian literature includes a cohort of infants with a 41.6% prevalence [35], a case series reporting a 50% prevalence among children with delayed CF diagnosis [62], and a Brazilian case report of severe HHMA during the summer [63]. Although these studies cannot establish a causal relationship between climate and HHMA, they provide convergent evidence that warm environmental conditions may represent an important contextual factor in susceptible individuals. The present study extends these observations by demonstrating HHMA in a larger CF cohort, including recurrent episodes and a clear seasonal pattern, while also providing evidence relevant to the pre-modulator era. Continued clinical surveillance, preventive electrolyte management, and timely recognition of salt-wasting episodes remain important components of CF care, particularly in regions characterized by high environmental temperatures.

### Limitations

This study has several limitations that should be considered when interpreting its findings. First, its retrospective design and reliance on medical records may have resulted in incomplete or inconsistently documented clinical, laboratory, and treatment-related information. Second, the study was conducted at a single specialized CF referral center, which may limit the generalizability of the findings to the broader CF population in Brazil and other healthcare settings. Third, although the overall cohort included 257 individuals with CF, only 41 patients met the criteria for HHMA. Moreover, recurrent episodes occurred in a relatively small proportion of these patients, substantially limiting the statistical power to perform reliable comparisons between recurrent and non-recurrent cases. Therefore, the observed relationship between the F508del genotype and recurrence should be interpreted as an exploratory finding rather than as evidence of a statistically established association.

Another limitation is the descriptive nature of the statistical analysis, which precluded robust adjustment for potential confounding factors and prevented the assessment of independent predictors of HHMA or recurrence. In addition, the odds ratios presented in Table 3 are not adjusted for multiple comparisons and should therefore be interpreted cautiously, particularly given the number of genetic comparisons performed. The observed seasonal pattern should likewise be interpreted cautiously, as environmental temperature, hydration status, intercurrent illness, nutritional status, sodium intake, pancreatic insufficiency, and other clinical factors may interact and could not be comprehensively controlled for in the retrospective dataset. Accordingly, the study cannot establish a causal relationship between environmental conditions and HHMA occurrence.

In addition, although CF diagnosis was confirmed by at least two sweat chloride measurements above 60 mEq/L, the exact sweat chloride values at diagnosis were not available in the database, precluding an assessment of their potential relationship with HHMA occurrence or severity. Baseline pancreatic insufficiency status was also not systematically available and could not be reliably evaluated as a potential clinical factor associated with HHMA. These limitations are particularly relevant because both the degree of CFTR dysfunction and pancreatic phenotype may influence the clinical expression of salt-wasting episodes.

The revised study includes a comparative analysis of pathogenic *CFTR* variants between patients with HHMA and the broader CF cohort followed at our reference center, providing a more detailed assessment of the genetic profile of both groups. However, a comprehensive comparison of all clinical characteristics between patients with and without HHMA was not possible because several variables were incompletely documented or unavailable for the broader cohort. Furthermore, the relatively small number of HHMA cases limits the ability to draw definitive genotype–phenotype conclusions for individual *CFTR* variants.

Finally, the study’s retrospective and single-center nature, together with the limited number of recurrent cases, restricts the ability to identify independent clinical, environmental, or genetic predictors of HHMA recurrence. Future multicenter studies with larger cohorts and prospective follow-up should evaluate these factors systematically and should also investigate whether contemporary CFTR-modulator therapies modify the incidence, recurrence, or severity of HHMA.

## 4. Materials and Methods

An analytical, retrospective study was conducted at a university-based CF referral center, including 257 individuals with CF followed between January 2014 and January 2024. All consecutive HHMA cases in the center during the study period were included. Data were obtained from medical records and an institutional database, including demographic, clinical, genetic, laboratory, microbiological, seasonal, and treatment-related variables.

CF diagnosis was established based on compatible clinical features and/or family history, associated with two sweat chloride tests ≥ 60 mEq/L and/or identification of two pathogenic *CFTR* variants [77,78,79]. HHMA was defined by the presence of: (i) primary metabolic alkalosis (pH ≥ 7.45 and bicarbonate ≥ 24 mmol/L); (ii) hyponatremia (≤135 mmol/L); (iii) hypochloremia (≤99 mmol/L); and (iv) hypokalemia (≤3.5 mmol/L) [13]. The classification of metabolic alkalosis was based on the concomitant elevation of pH and bicarbonate, together with the characteristic clinical context of salt and fluid loss. Respiratory compensation was considered an expected physiological response to metabolic alkalosis and was not considered evidence against the diagnosis of HHMA. A primary respiratory acid–base disorder was not considered when there was no clinical or laboratory evidence suggesting an independent respiratory disturbance. When blood gas measurements were available, the acid–base profile was interpreted in the context of the expected respiratory compensation for metabolic alkalosis rather than as a criterion for excluding HHMA.

A total of 41 patients met the diagnostic criteria and were included in the analysis. Variables collected included sex, age at first HHMA episode, clinical manifestations, newborn screening results, *CFTR* genotype, recurrence, seasonality, laboratory parameters (pH, serum sodium, serum potassium, serum chloride, and serum bicarbonate), microbiological profile, and treatment strategies. All electrolyte and acid–base measurements included in the analysis were obtained at the time of HHMA diagnosis and before the initiation of intravenous or oral fluid, electrolyte, or other specific treatment for the episode.

Regarding the temporal relationship between HHMA and CF diagnosis, all patients who had undergone neonatal screening had received this screening before the occurrence of HHMA. Therefore, in these patients, HHMA was not the clinical finding that led to the diagnosis of CF. For participants without documented neonatal screening, the available records did not allow reliable retrospective determination of whether HHMA preceded the diagnosis of CF.

The *CFTR* gene genotype was identified as previously described [16,17]. *CFTR* variants were assigned to functional classes (Classes I–VI) according to the traditional *CFTR* variant classification system, based on their established or reported effects on CFTR protein production, processing, regulation, conductance, synthesis, and stability, as described in the published literature [15,16,17,18,19,20,21,22,23,24,25,26,27,28].

Class I variants are characterized by absent or severely defective CFTR protein production, typically resulting from premature termination, abnormal splicing, or other mechanisms that prevent the production of functional protein. Class II variants result in defective CFTR protein processing, folding, and maturation, leading to impaired trafficking of the protein to the cell surface. Class III variants are associated with defective CFTR regulation or gating, resulting in impaired channel activation despite the presence of CFTR at the cell membrane. Class IV variants cause defective chloride conductance through the CFTR channel, with reduced ion transport despite relatively preserved protein expression and channel regulation. Class V variants are associated with reduced CFTR protein synthesis, generally resulting in decreased amounts of functional CFTR at the cell surface. Class VI variants are characterized by reduced CFTR protein stability, leading to increased turnover and a shortened residence time of functional CFTR at the cell membrane. When the available evidence was insufficient to assign a variant reliably to one of these functional classes, the variant was categorized as not classified.

Data were analyzed using descriptive statistics, with continuous variables summarized as median (interquartile range) and categorical variables expressed as absolute frequencies and percentages [n (%)]. Comparisons between patients with and without HHMA were performed using Pearson’s chi-square test or Fisher’s exact test, as appropriate. Odds ratios (ORs) and 95% confidence intervals (95% CIs) were calculated to estimate the strength and precision of associations, with patients without HHMA considered the reference group. For the F508del genotype analysis, the —/— genotype was used as the reference category. For grouped *CFTR* genotype and sex analyses, the second category of each variable was used as the reference (Table 3). Statistical analyses and graphical representations were performed using GraphPad Prism (version 10.5.0; GraphPad Software, San Diego, CA, USA) and IBM SPSS (Statistical Package for the Social Sciences) Statistics (version 26; IBM Corp., Armonk, NY, USA). All tests were two-sided, and a *p*-value < 0.05 was considered statistically significant.

The study was approved by the Research Ethics Committee of the University of Campinas (No. 43787320.100000.5404), in accordance with Resolution No. 466/12 of the National Health Council. Written informed consent was obtained from all individual participants included in the study or from their legal guardians in the case of minors or individuals unable to provide consent.

## 5. Conclusions

Our findings highlight that HHMA is a frequent, early, and potentially recurrent complication of CF, strongly influenced by genotype and environmental factors, and should be systematically considered in clinical practice to improve early diagnosis and management.

## Figures and Tables

**Table 1:** Cystic Fibrosis Transmembrane Conductance Regulator (*CFTR*) genotypic and variant-class profile among patients with cystic fibrosis and hypokalemic hypochloremic metabolic alkalosis (HHMA) at a Brazilian referral center.

Patient	Genotype	*CFTR* Variant Class
1	F508del/R1162X	II/I
2	F508del/S4X	II/I
3	F508del/S4X	II/I
4	G542X/Q1100P	I/II
5	F508del/S549R	II/III
6	F508del/A561E	II/II
7	F508del/G85E	II/II
8	G542X/A561E	I/II
9	F508del/G542X	II/I
10	F508del/CFTRdele14b and CFTRdup9	II/I
11	F508del/2184del_A	II/I
12	F508del/G542X	II/I
13	F508del/G542X	II/I
14	F508del/F508del	II/II
15	R1162X/D614G	I/II
16	S466X;R1070Q/G542X	I;I/I
17	F508del/F508del	II/II
18	G542X/1812-1G>A	I/I
19	F508del/F508del	II/II
20	F508del/621+1G>T	II/I
21	F508del/R1162X	II/I
22	F508del/Q1100P	II/II
23	F508del/3120+1G>A	II/I
24	G542X/S549R	I/III
25	N1303K/N1303K	II/II
26	F508del/G85E	II/II
27	N1303K/3120+1G>A	II/I
28	F508del/F508del	II/II
29	F508del/F508del	II/II
30	G542X/N1303K	I/II
31	F508del/F508del	II/II
32	F508del/F508del	II/II
33	IVS8-5T/W1098C	V/NC
34	F508del/875+1G>A	II/I
35	F508del/S446X	II/I
36	F508del/R1066C	II/III
37	F508del/R334W	II/IV
38	F508del/F508del	II/II
39	F508del/Y1092X	II/I
40	3617delGA/3905insT	I/I
41	F508del/F508del	II/II

*CFTR* variant classes were assigned according to the traditional functional classification (Classes I–VI) based on established literature. Variants for which a classification could not be established with sufficient certainty were left unclassified. Class I: defective protein production; Class II: defective processing and maturation; Class III: defective regulation/gating; Class IV: defective conductance; Class V: reduced CFTR synthesis; Class VI: reduced protein stability [15,16,17,18,19,20,21,22,23,24,25,26,27,28]; NC: not classified. Class VI variants were not identified in any participant in the present study.

**Table 2:** Cystic Fibrosis Transmembrane Conductance Regulator (*CFTR*) genotypic and variant-class profile among patients with cystic fibrosis without hypokalemic hypochloremic metabolic alkalosis (HHMA) at a reference center in Brazil.

Genotype	*CFTR* Variant Class	Number of Cases
—/—	—/—	12
2183AA>G/2183AA>G	I/I	1
2752-26A>G/2752-26A>G	V/V	1
3120+1G>A/—	I/—	1
3617delGA/3905insT	—/—	1
4006-1G>A/3272-26A>G	I/V	1
A561E/A561E	II/II	1
A561E/Y913X	II/I	1
F508del/CFTRdele7-18	II/I	2
F508del/1716+18672A>G	II/V	2
F508del/1717-1A>G	II/I	2
F508del/—	II/—	6
F508del/1812-1G>A	II/I	2
F508del/2183AA>G	II/I	4
F508del/2184ins_A	II/I	1
F508del/2556insAT	II/I	1
F508del/2789+5G>A	II/IV	3
F508del/3120+1G>A	II/I	5
F508del/3272-26A>G	II/V	2
F508del/3849+10kbc>T	II/V	1
F508del/621+1G>C	II/I	1
F508del/711+1G>T	II/I	1
F508del/A559T	II/II	1
F508del/A561E	II/II	1
F508del/D1152H	II/IV	1
F508del/CFTRdele9	II/I	1
F508del/CFTRdele16-20	II/I	2
F508del/CFTRdup7-11	II/I	1
F508del/F508del	II/II	68
F508del/G542X	II/I	17
F508del/G576A	II/NC	1
F508del/G646X	II/I	1
F508del/G85E	II/II	2
F508del/G85V	II/II	1
F508del/L365P	II/NC	1
F508del/N1303K	II/II	4
F508del/P205S	II/IV	4
F508del/P5L	II/NC	1
F508del/Q1100P	II/II	1
F508del/Q1238X	II/I	1
F508del/Q552X	II/I	1
F508del/Q890X	II/I	1
F508del/R1066C	II/II	2
F508del/R1162X	II/II	1
F508del/R334W	II/IV	4
F508del/R347P	II/IV	1
F508del/S1235R	II/NC	1
F508del/S4X	II/I	7
F508del/T291I	II/NC	1
F508del/V874E	II/NC	1
F508del/W1310X	II/I	1
F508del/V232D	II/II	1
F508del/Y1092X	II/I	1
G542X/—	I/—	2
G542X/1812-1G>A	I/I	1
G542X/2556insAT	I/I	1
G542X/3120+1G>A	I/I	1
G542X/711+1G>T	I/I	1
G542X/G542X	I/I	1
G542X/G576A;R668C	I/NC	1
G542X/I618T	I/IV	1
G542X/Q1100P	I/II	1
G542X/R1066C	I/II	3
G542X/R1162X	I/I	2
G542X/R334W	I/IV	1
G542X/S4X	I/I	1
G542X/S549R	I/III	1
N1303K/2183AA>G	II/I	1
Q552X/S4X	I/I	1
R1162X/N1303K	I/II	1
R1162X/R1162X	I/I	2
R258G/—	NC/—	1
R74W;D1270N/—	NC/—	1
S1235R/—	NC/—	1
S466X/A561E	I/II	1
S466X;R1070Q/A561E	I;I/II	1
S4X/N1303K	I/II	1
S4X/S549R	I/III	1
W1274X/—	I/—	1
Y1092X/N1303K	I/II	1

*CFTR* variant classes were assigned according to the traditional functional classification (Classes I–VI) based on established literature. Variants for which a classification could not be established with sufficient certainty were left unclassified. Class I: defective protein production; Class II: defective processing and maturation; Class III: defective regulation/gating; Class IV: defective conductance; Class V: reduced CFTR synthesis; Class VI: reduced protein stability [15,16,17,18,19,20,21,22,23,24,25,26,27,28]; —: not informed; NC: not classified. Class VI variants were not identified in any participant in the present study.

**Table 3:** Comparison of Cystic Fibrosis Transmembrane Conductance Regulator (*CFTR*) genotype profile and sex between patients with and without hypokalemic hypochloremic metabolic alkalosis (HHMA).

Group	Patients with HHMA	Patients Without HHMA	Total	*p*-Value	OR (95% CI)
F508del genotype					
F508del/F508del	9 (22.0%)	68 (31.5%)	77 (30.0%)	0.340	0.614 (0.237–1.591)
F508del/—	21 (51.2%)	97 (44.9%)	118 (45.9%)	1.000	1.004 (0.449–2.244)
—/—	11 (26.8%)	51 (23.6%)	62 (24.1%)		Reference
Grouped *CFTR* genotype					
Two *CFTR* variants from I, II, and/or III class	39 (95.1%)	160 (74.1%)	199 (77.4%)	0.002	6.83 (1.60–29.19)
At least one *CFTR* variant from classes III, IV, and/or VI, or one/two unclassified/not identified variants	2 (4.9%)	56 (25.9%)	58 (22.6%)		Reference
Sex					
Male	29 (70.7%)	106 (49.1%)	135 (52.5%)	0.016	2.51 (1.22–5.17)
Female	12 (29.3%)	110 (50.9%)	122 (47.5%)		Reference

Data are presented as number of patients and percentage [n (%)]. Categorical variables were compared using Pearson’s chi-square test or Fisher’s exact test, as appropriate. Odds ratios (ORs) and 95% confidence intervals (95% CIs) were calculated using the group without HHMA as the reference. For F508del genotype, the —/— genotype was used as the reference category. For grouped *CFTR* genotype and sex, the second category shown was used as the reference. The symbol “—” indicates that the second allele is different from F508del and may correspond to another *CFTR* variant or, in rare cases, to no identifiable variant.

## Data Availability

The material used to conduct this study is available upon request from the corresponding authors.

## Supplementary Materials

The following supporting information can be downloaded at https://www.mdpi.com/article/10.3390/ijms27177668/s1.

## Abbreviations

The following abbreviations are used in this manuscript:

±	Plus or minus
%	Percentage
≥	Greater than or equal to
≤	Less than or equal to
95% CI	95% confidence interval
CA	California
CF	Cystic fibrosis
CFTR	Cystic Fibrosis Transmembrane Conductance Regulator
Cl^−^	Chloride
CLCNKB	Chloride Voltage-Gated Channel Kb
CO_2_	Carbon dioxide
d	Days
Fem	Female
H^+^	Hydrogen ion
HCO_3_^−^	Bicarbonate
HHMA	Hypokalemic hypochloremic metabolic alkalosis
HSD11B2	Hydroxysteroid 11-Beta Dehydrogenase 2
IQR	Interquartile range
K^+^	Potassium
Kg	kilogram
m	Months
MCT	Medium-Chain Triglycerides
mEq/L	Milliequivalents per liter
MG	Minas Gerais
mmol/L	Millimoles per liter
N	Number of individuals
Na^+^	Sodium
NaCl	Sodium chloride
NC	Not confirmed
NBS	Newborn screening
NR	Not reported
NY	New York
OMIM	Online Mendelian Inheritance in Man
OR	Odds ratio
pH	Hydrogen potential
PUC-Campinas	Pontifícia Universidade Católica de Campinas
Ref	Reference
RNA	Ribonucleic acid
SD	Standard deviation
SLC26A3	Solute Carrier Family 26 Member 3
SLC26A4	Solute Carrier Family 26 Member 4
SP	São Paulo
SPSS	Statistical Package for the Social Sciences
SUS	Sistema Único de Saúde
Unicamp	Universidade de Campinas
Univás	Universidade do Vale do Sapucaí
USA	United States of America
USF	Universidade São Francisco
y	Years
