# Supplementary material for: Hypokalemic Hypochloremic Metabolic Alkalosis in Cystic Fibrosis: A Retrospective Observational Study with Clinical Characterization, CFTR Genetic Profiling, and a Comparative Genetic Analysis with Patients from a Brazilian Reference Center
Source: Int J Mol Sci. 2026 Aug 27;27(17):7668. doi: 10.3390/ijms27177668 (PMC13566040; doi:10.3390/ijms27177668)
Supplement: Supplementary file 1 [file ijms-27-07668-s001.zip › ijms-4454715-supplementary.pdf]

Supplementary Table S1. Clinical characteristics, cystic fibrosis diagnostic features, and seasonality of hypokalemic hypochloremic metabolic alkalosis (HHMA) in individuals with cystic fibrosis.

Patient	Sex	NBS	Sweat test	Age at 1st HHMA episode	Symptoms	Seasonality at 1st HHMA episode
1	Fem	Altered	Altered	4m 5d	Hypoactivity, reduced oral intake	Summer
2	Fem	Altered	Altered	26d	Diarrhea, vomiting	Summer
3	Male	Altered	Altered	4m 25d	Dehydration, malnutrition	Winter
4	Male	Altered	Altered	5m 8d	Dehydration	Fall
5	Fem	NR	Altered	5m 22d	Asymptomatic	Summer
6	Male	Altered	Altered	3m 5d	Dehydration	Winter
7	Fem	Altered	Altered	2m 27d	Dehydration, malnutrition	Spring
8	Male	NR	Altered	13y	Dehydration, vomiting	Spring
9	Male	Altered	Altered	4m 8d	Dehydration, reduced oral intake	Summer
10	Fem	Altered	Altered	3m 7d	Cough, fever, malnutrition	Winter
11	Male	Altered	Altered	2m 7d	Dehydration	Summer
12	Male	NR	Altered	3m 28d	Dehydration, cough, fever, vomiting	Summer
13	Male	NR	Altered	5m 23d	Dehydration	Spring
14	Male	Altered	Altered	3m 13d	Dehydration, cough, hypoactivity	Summer
15	Male	Normal	Altered	3m 22d	Fever, cough, reduced oral intake	Summer
16	Male	NR	Altered	1m 20d	Dehydration, reduced oral intake	Fall
17	Male	NR	Altered	1m 29d	Dehydration, weight loss, diarrhea	Fall
18	Fem	Altered	Altered	3m 27d	Dehydration, weight loss, vomiting, cough	Summer
19	Male	Altered	Altered	10m 15d	Dehydration, vomiting, diarrhea	Summer
20	Male	Altered	Altered	5m 22d	Reduced oral intake, cough, dehydration	Summer
21	Male	Altered	Altered	4m 13d	Cough, fever, dehydration	Winter
22	Male	Altered	Altered	4m	Dehydration, weight loss	Spring
23	Male	Altered	Altered	4m	Dehydration, vomiting, diarrhea	Summer
24	Fem	Altered	Altered	5m 17d	Asymptomatic	Summer
25	Fem	Altered	Altered	3m 6d	Vomiting, diarrhea, hypoactivity, cough	Spring
26	Fem	Altered	Altered	3m 16d	Dehydration	Summer
27	Male	Altered	Altered	2y 4m	Dehydration, hypoactivity	Winter
28	Male	Altered	Altered	4m 6d	Dehydration	Fall
29	Male	Altered	Altered	4m 20d	Reduced oral intake, hypoactivity	Summer
30	Male	Altered	Altered	1y 6m	Reduced oral intake	Summer
31	Fem	Altered	Altered	1m 22d	Asymptomatic	Fall
32	Fem	Altered	Altered	10m	Fever, cough, dehydration	Summer
33	Male	Normal	Altered	3m 21d	Cough, dehydration, hypoactivity, reduced oral intake	Summer
34	Male	Normal	Altered	3m 28d	Dehydration, irritability	Spring
35	Male	Altered	Altered	4m 6d	Dehydration, hypoactivity, cough, reduced oral intake, weight loss	Spring
36	Male	Altered	Altered	8m 24d	Hypoactivity, vomiting, nasal obstruction	Spring
37	Male	Altered	Altered	5m 29d	Dehydration, malnutrition	Summer
38	Male	Altered	Altered	1y 11m	Reduced oral intake, dehydration, vomiting, diarrhea	Summer

Patient	Sex	NBS	Sweat test	Age at 1st HHMA episode	Symptoms	Seasonality at 1st HHMA episode
39	Male	Normal	Altered	1y 1m	Dehydration	Spring
40	Male	Normal	Altered	5y	Vomiting, reduced oral intake, hypoactivity	Summer
41	Fem	NR	Altered	2m 10d	Dehydration, vomiting, diarrhea, malnutrition	Spring

d: days, Fem: female, m: months, NBS: newborn screening, NR: not reported; y: years.

Supplementary Table S2. Laboratory values of pH, sodium, potassium, chloride, and bicarbonate, as well as the profile of bacterial colonization (*Staphylococcus aureus*, *Pseudomonas aeruginosa*, and *Moraxella catarrhalis*) prior to diagnostic of hypokalemic hypochloremic metabolic alkalosis (HHMA) in individuals with cystic fibrosis.

Patient	pH	Na^+	K^+	Cl^-	HCO_3^-	Colonization prior to HHMA diagnosis
1	7.67	116	2.7	63	41.9	*S. aureus*
2	7.48	127	2.6	90	18.6	*S. aureus*
3	7.46	126	3.3	86	40.5	NR
4	7.46	127	2.7	82	NR	*S. aureus* and *P. aeruginosa*
5	7.44	122	3.0	80	36.3	NR
6	7.58	134	3.1	93	29.8	*S. aureus*
7	7.48	127	2.6	90	27.9	NR
8	7.49	135	3.2	98	24.7	NR
9	7.46	131	2.3	93	31.2	*S. aureus*
10	7.66	113	2.3	65	52.2	NR
11	7.56	122	2.7	82	32.9	*S. aureus*
12	7.62	122	2.6	79	37.0	*P. aeruginosa*
13	7.55	126	2.6	76	46.0	*S. aureus*
14	7.50	111	3.6	61	37.2	NR
15	7.59	128	1.7	61	64.8	*S. aureus* and *P. aeruginosa*
16	7.49	131	3.2	86	36.1	NR
17	7.52	130	4.5	89	28.7	*S. aureus* and *P. aeruginosa*
18	7.49	132	2.9	91	32.4	*S. aureus*
19	7.49	134	2.6	94	29.5	*P. aeruginosa*
20	7.55	118	2.9	NR	44.0	*S. aureus*
21	7.67	114	2.2	64	45.1	*S. aureus*
22	7.45	125	4.2	86	26.2	*S. aureus* and *P. aeruginosa*
23	7.51	119	3.0	72	36.6	*S. aureus*
24	7.54	123	2.8	76	40.7	*S. aureus* and *P. aeruginosa*
25	7.48	113	2.4	77	37.4	NR
26	7.50	126	3.1	87	31.7	*S. aureus* and *P. aeruginosa*
27	7.46	131	2.6	95	29.5	*S. aureus* and *P. aeruginosa*
28	7.60	127	3.3	92	24.8	*S. aureus*
29	7.53	122	2.7	66	45.5	*S. aureus* and *P. aeruginosa*
30	7.46	133	2.7	91	34.1	*S. aureus* and *P. aeruginosa*
31	7.52	125	4.0	82	37.1	*S. aureus*
32	7.47	132	3.1	94	29.4	NR
33	7.53	125	2.6	70	44.2	NR
34	7.63	132	3.4	84	34.4	NR
35	7.55	125	3.0	76	38.5	*S. aureus*
36	7.68	106	2.3	49	45.6	*S. aureus* and *P. aeruginosa*
37	7.47	132	3.5	98	34.0	*S. aureus*
38	7.50	131	3.3	88	29.4	NR

Patient	pH	Na^+	K^+	Cl^-	HCO_3^-	Colonization prior to HHMA diagnosis
39	7.58	114	2.1	58	44.5	*S. aureus*, *E. coli*, and *M. catarrhalis*
40	7.80	124	2.0	67	44.4	*S. aureus*
41	7.56	125	1.9	76	47.6	NR

Cl^-: chloride, HCO_3^-, bicarbonate, K^+: potassium, Na^+: sodium, NR: not reported, pH: hydrogen potential (measure of acidity or alkalinity). HHMA was defined by the presence of: (i) primary metabolic alkalosis (pH ≥ 7.45 and bicarbonate ≥ 24 mmol/L) in the absence of respiratory compensation; (ii) hyponatremia (≤ 135 mmol/L); (iii) hypochloremia (≤ 99 mmol/L); and (iv) hypokalemia (≤ 3.5 mmol/L) [13]. mmol/L: millimoles per liter.

Supplementary Table S3. Treatment administered, age at recurrence, and seasonality of hypokalemic hypochloremic metabolic alkalosis (HHMA) in individuals with cystic fibrosis.

Patient	Treatment	Recurrence	Seasonality
1	Intravenous hydration, oral hydration, electrolyte replacement	No	No
2	NR (external admission)	No	No
3	Intravenous hydration, oral hydration, electrolyte replacement, orotracheal intubation	No	No
4	Intravenous hydration, oral hydration, electrolyte replacement	No	No
5	Intravenous hydration, oral hydration, electrolyte replacement	1 (6m 26d)	Summer
6	Intravenous hydration, oral hydration, electrolyte replacement	1 (4m 15d)	Spring
7	Intravenous hydration, oral hydration, electrolyte replacement	No	No
8	Intravenous hydration	No	No
9	Intravenous hydration, oral hydration, electrolyte replacement	No	No
10	Intravenous hydration, oral hydration, electrolyte replacement, tent, nasal cannula	No	No
11	Intravenous hydration, oral hydration, electrolyte replacement	2 (2m 14d and 2m 23d)	Summer and summer
12	NR (external admission)	No	No
13	Intravenous hydration, oral hydration, electrolyte replacement	No	No
14	Intravenous hydration, oral hydration, electrolyte replacement, orotracheal intubation, antibiotic therapy	No	No
15	Intravenous hydration, oral hydration, electrolyte replacement, antibiotic therapy	No	No
16	NR (external admission)	No	No
17	Intravenous hydration, oral hydration, electrolyte replacement, nasogastric tube (nutrition)	3 (2m 11d, 9m 18d, and 3y 9m)	Fall, summer, and summer
18	Intravenous hydration, oral hydration, electrolyte replacement, antibiotic therapy	No	No
19	Intravenous hydration, electrolyte replacement	No	No
20	Intravenous hydration, oral hydration, electrolyte replacement, orotracheal intubation, antibiotic therapy	No	No
21	Intravenous hydration, oral hydration, electrolyte replacement, antibiotic therapy	No	No
22	Oral hydration	1 (5m)	Spring
23	Intravenous hydration	1 (1y 4m)	Spring
24	Intravenous hydration, oral hydration, electrolyte replacement	No	No
25	Intravenous hydration, oral hydration, electrolyte replacement	1 (1y 3m)	Spring
26	Oxygen tent, intravenous hydration, oral hydration, fenoterol	No	No
27	Intravenous hydration, electrolyte replacement, antibiotic therapy	No	No
28	Intravenous hydration, oral hydration, electrolyte replacement	No	No
29	Intravenous hydration, oral hydration, electrolyte replacement	No	No
30	Intravenous hydration, oral hydration, electrolyte replacement	No	No
31	Intravenous hydration, oral hydration, electrolyte replacement	No	No

32	Oral hydration, antibiotic therapy	No	No
33	Intravenous hydration, electrolyte replacement, antibiotic therapy	3 (4m 18d, 5m 11d, and 5m 22d)	Summer, summer, and summer
34	Intravenous hydration, oral hydration	No	No
35	Intravenous hydration, oral hydration, electrolyte replacement, tent, nasal cannula	No	No
36	Intravenous hydration, oral hydration, electrolyte replacement	1 (1y)	Summer
37	Intravenous hydration, oral hydration, electrolyte replacement, parenteral nutrition, tent, nasal cannula, vitamin K, antibiotic therapy	2 (1y 2m and 1y 4m)	Fall and summer
38	Intravenous hydration	No	No
39	Oral hydration, salbultamol, dornase alfa	No	No
40	NR (external admission)	No	No
41	Intravenous hydration, oral hydration, electrolyte replacement	2 (3m 3d and 4m 17d)	Spring and summer

d: days, m: months, NR: not reported, y: years.

Supplementary Table S4. Summary of published studies on hypokalemic hypochloremic metabolic alkalosis (HHMA) associated with cystic fibrosis including countries, journals, and study designs.

Ref	Title	Country	Journal	Study design
[7]	Cystic fibrosis presenting as pseudo-Bartter syndrome: An important diagnosis that is missed!	India	The Indian Journal of Pediatrics	Case report
[57]	The success of the Cystic Fibrosis Registry of Turkey for improvement of patient care	Turkey	Pediatric Pulmonology	Observational
[31]	Characteristics of electrolyte imbalance and pseudo-Bartter syndrome in hospitalized cystic fibrosis children and adolescents	Pakistan	Journal of Cystic Fibrosis	Observational
[58]	Clinical features and accompanying findings of pseudo-Bartter syndrome in cystic fibrosis	Turkey	Pediatric Pulmonology	Observational
[35]	Uncommon presentation of cystic fibrosis: A case report and literature review	Israel	Cureus	Case report
[36]	Pseudo-Bartter syndrome and intermediate sweat chloride levels-it could still be cystic fibrosis!	India	Indian Journal of Pediatrics	Case report
[30]	Cystic fibrosis in Turkey: First data from the national registry	Turkey	Pediatric Pulmonology	Observational
[59]	Should isolated pseudo-Bartter syndrome be considered a CFTR-related disorder of infancy?	Italy	Pediatric Pulmonology	Observational
[32]	Prevalence and clinical features of cystic fibrosis with pseudo-Bartter syndrome	Turkey	Annals of Tropical Paediatrics	Observational
[60]	An epidemic of pseudo- Bartter syndrome in cystic fibrosis patients	Turkey	European Journal of Pediatrics	Observational
[13]	Clinical features and treatment approaches in cystic fibrosis with pseudo-Bartter syndrome	Turkey	Annals of Tropical Paediatrics	Observational
[55]	Mimicry and well-known genetic friends: molecular diagnosis in an Iranian cohort of suspected Bartter syndrome and proposition of an algorithm for clinical differential diagnosis	Iran	Orphanet Journal of Rare Diseases	Case report
[37]	Cystic fibrosis in Saudi Arabia: common and rare presentations	Saudi Arabia	Annals of Tropical Paediatrics	Case report
[61]	Cystic fibrosis in Arabs: a prototype from Jordan	Jordan	Annals of Tropical Paediatrics	Observational
[10]	Pseudo-Bartter's syndrome in cystic fibrosis	United Kingdom	Archives of Disease in Childhood	Case report
[38]	Metabolic alkalosis with hypo-electrolytaemia or pseudo-Bartter syndrome as a presentation of cystic fibrosis in infancy. Description of three cases	Spain	European Journal of Pediatrics	Case report
[39]	Hot summer shines light on diagnosis of Pseudo-Bartter Syndrome	India	The Indian Journal of Pediatrics	Case report
[11]	Pseudo-Bartter syndrome, pattern and correlation with other cystic fibrosis features	Jordan	Saudi Journal of Kidney Diseases and Transplantation	Observational

Ref	Title	Country	Journal	Study design
[33]	Elevated sweat potassium, hyperaldosteronism and pseudo-Bartter's syndrome: A spectrum of disorders associated with cystic fibrosis	England	Journal of the Royal Society of Medicine	Observational
[8]	Pseudo-Bartter as an initial presentation of cystic fibrosis. A case report and review of the literature	United Arab Emirates	Eastern Mediterranean Health Journal	Case report
[14]	Episodic seasonal pseudo-Bartter syndrome in cystic fibrosis	England	Paediatric Respiratory Reviews	Case report
[34]	Prevalence and nutritional status of infants with cystic fibrosis and pseudo-Bartter syndrome during the first year of life	Brazil	Infant, Child, & Adolescent Nutrition	Observational
[40]	Pseudo-Bartter syndrome in children with cystic fibrosis	Iran	Clinical Case Report	Case report
[52]	Pseudo-Bartter syndrome as the sole manifestation of cystic fibrosis in a child with 711+G>T/IVS8-5T mutation: a new face of an old disease	Tunisia	Annales de Biologie Clinique	Case report
[41]	Vomiting may be the only sign of cystic fibrosis: a case report	Turkey	Gazi Medical Journal	Case report
[42]	Dehydrated patient without clinically evident cause: A case report	Italy	World Journal of Clinical Cases	Case report
[12]	Pseudo-Bartter's syndrome in patients with cystic fibrosis: A case series and review of the literature	Serbia	Serbian Archives of Entire Medicine	Case report
[43]	Case of pseudo-Bartter's syndrome: an atypical presentation of cystic fibrosis	Nepal	Journal of Nepal Paediatric Society	Case report
[44]	Profound electrolyte and acid-base disturbances in an infant with cystic fibrosis	USA	Pediatric Pulmonology	Case report
[9]	Pseudo-Bartter syndrome in Chinese children with cystic fibrosis: Clinical features and genotypic findings	China	Pediatric Pulmonology	Case report
[45]	A difficult case of hyponatremic and hypokalemic metabolic alkalosis: Answers	Italy	Pediatric Nephrology	Case report
[46]	Pseudo-Bartter's syndrome revealing cystic fibrosis in an infant caused by 3849 + 1G>A and 4382delA compound heterozygosity	Lebanon	Acta Pædiatrica	Case report
[47]	Pseudo-Bartter syndrome in a Chinese infant with cystic fibrosis caused by c.532G>A mutation in *CFTR*	China	Chinese Medical Journal	Case report
[48]	Cystic fibrosis mimicking Bartter's syndrome	United Kingdom	Acta Paediatrica Scandinavica	Case report
[49]	Normal sweat chloride test does not rule out cystic fibrosis	Turkey	Turkish Journal of Pediatrics	Case report
[50]	K162E – A rare and uncategorized *CFTR* variant causing cystic fibrosis	Brazil	Journal of Cystic Fibrosis	Case report
[54]	The Case | Metabolic alkalosis in a patient with cystic fibrosis	USA	Kidney International	Case report
[53]	Metabolic alkalosis with multiple salt unbalance: An atypical onset of cystic fibrosis in a child	Italy	Journal of Pediatric and Neonatal Individualized Medicine	Case report

Ref	Title	Country	Journal	Study design
[51]	Atypical presentation of cystic fibrosis-obese adolescent with hypertension and pseudo-Bartter's syndrome	Serbia	Vojnosanitetski Pregled	Case report
[56]	Modified oral rehydration therapy in a case with cystic fibrosis	Turkey	The Turkish Journal of Pediatrics	Case report
[29]	Diverse genotypical features and impacts on clinical course and severity of cystic fibrosis: early childhood experience	Turkey	Minerva Pediatrica	Case report

Ref: reference, USA: United States of America.

Supplementary Table S5. Study objectives and clinical, genetic, and outcome characteristics of published studies on hypokalemic hypochloremic metabolic alkalosis (HHMA) in individuals with cystic fibrosis (CF).

Ref	Sample	Objective	Clinical presentation	Genetics	Outcome	Conclusion
[7]	n = 1, female infant, four months old.	Discuss HHMA as an initial manifestation of CF, highlighting the importance of differentiating it from Bartter syndrome, Gitelman syndrome, and other differential diagnoses.	Growth failure, recurrent dehydration, and an episode of pneumonia. Laboratory findings showed hyponatremia, hypokalemia, hypochloremia, and metabolic alkalosis. The child had severe malnutrition.	F508del/F508del.	Confirmation of CF diagnosis through sweat chloride testing and genetic analysis. Correction of electrolyte imbalances was performed, with clinical improvement following appropriate management.	HHMA can be the initial manifestation of CF. It is essential to consider CF in the differential diagnosis of hypokalemia and metabolic alkalosis in infants to avoid delays in diagnosis and treatment.
[57]	n = 2,807 (85 with HHMA).	To evaluate the association between the national CF registry system and improvements in patient follow-up, growth, treatments, and complications.	Not reported.	No specific genetic data were reported for this sample.	Improvement in height and body mass index z-scores. Increased use of medications that enhance lung function, such as hypertonic saline solution, bronchodilators, and azithromycin. Reduction in the incidence of HHMA, attributed to increased awareness provided by the national registry. Regular salt supplementation is recommended, especially for patients in hot climate regions and children vulnerable to fluid loss. Increased frequency of CF-related complications, such as liver disease and osteoporosis.	The national CF registry system in Turkey increased awareness and improved patient follow-up care. The widespread adoption of the system is promising for the clinical management of the CF population in the country. Underreporting and the absence of newborn screening in some countries have been associated with a higher prevalence of HHMA, highlighting the role of national registries in the identification and management of the condition.
[31]	n = 72 (11 with HHMA) / mean age 3.81 ± 0.86	To determine and compare clinical and laboratory characteristics in children and	The study highlighted HHMA as a rare manifestation of CF, characterized by hypokalemic metabolic alkalosis,	The pathogenic F508del variant was identified in 14% of patients in the non-HHMA	HHMA was identified as the initial manifestation in 15% of cases. The mean age of patients with HHMA was	HHMA can be the initial manifestation in CF or occur at any time during the course of the disease. Anyone with

Ref	Sample	Objective	Clinical presentation	Genetics	Outcome	Conclusion
	years / age at diagnosis 2.34 ± 0.53 years.	adolescents with CF, highlighting HHMA and electrolyte imbalance for early diagnosis.	hyponatremia, and hypochloremia. Patients with HHMA exhibited a higher frequency of polyuria, gastrointestinal disorders (diarrhea, vomiting, and constipation), and younger age at diagnosis.	group, 16% in the exocrine insufficiency group, and 9% in the HHMA group.	lower (3.81 ± 0.86 years). Polyuria was prevalent in the HHMA group (72%). *Pseudomonas* colonization was more frequent in the non-HHMA groups.	CF who does not improve despite treatment should be evaluated for HHMA. In previously healthy children with hyponatremic hypokalemic metabolic alkalosis, CF with HHMA should be considered.
[58]	n = 1,170 (120 with HHMA) / mean age at diagnosis: 0.77 ± 1.7 years.	Compare clinical characteristics and findings of patients with and without HHMA within a population with CF.	HHMA is characterized by hyponatremic dehydration and hypochloremia with metabolic alkalosis, without renal disease. It is more common in young ages.	Variants F508del, N1303K, D110H, G542X, and E92K were identified.	HHMA was the most common complication, primarily affecting younger patients. No differences were observed between groups regarding genetic mutations or colonization.	HHMA is a common complication of CF in hot climates and can be an early diagnostic indicator in young patients.
[35]	n = 1 / five months.	To report a case of CF in an infant of African descent with HHMA as the initial manifestation and normal prenatal genetic screening.	Weight loss and dehydration without apparent cause. Presented hypokalemia, hyponatremia, and hypochloremia, as well as severe hypokalemic and hypochloremic metabolic alkalosis. No history of respiratory or gastrointestinal symptoms.	Rare pathogenic variant in the *CFTR* gene (H139L) identified by next-generation sequencing.	Diagnosis confirmed by sweat test and genetic sequencing. Treatment involved electrolyte replacement and referral for specialized follow-up.	HHMA can be an initial manifestation of CF, even in the absence of respiratory or gastrointestinal symptoms. This case underscores the need to consider CF regardless of ethnicity and prenatal screening.
[36]	n = 8; majority diagnosed before seven months of age, usually during the first summer.	Highlight the possibility of CF in infants with HHMA, even with intermediate sweat chloride levels.	Metabolic alkalosis, elevated bicarbonate, hyponatremia, and hypokalemia.	Pathogenic variants were identified in three patients, but they were not described in the study.	CF diagnosis was confirmed in all patients: five by repeat sweat testing and three by genetic analysis. The initial phenotype was milder, but within the first decade, three patients developed bronchiectasis and one died from respiratory failure.	The study reinforces that intermediate sweat chloride levels require follow-up and repeat testing for a reliable diagnosis.

Ref	Sample	Objective	Clinical presentation	Genetics	Outcome	Conclusion
[30]	n = 1,170 (120 with HHMA).	To describe and analyze the first data from the Turkish National CF Registry.	Clinical data from the Turkish National CF Registry, analyzing clinical, genetic, and therapeutic characteristics of the diagnosed population.	Genetic variants were not specified for patients with HHMA, only for the overall registry. The most common mutation was F508del (28% allele frequency). Other frequent mutations: N1303K (4.9%), G542X (4.5%), and 1677delTA (4.0%). In 25.2% mutations were not identified.	Challenges in obtaining complete genetic data and standardizing sweat tests. Need to increase inclusion of adult patients. Importance of newborn screening to reduce age at diagnosis.	The study highlights the challenges in implementing the registry and points to ways to improve CF care and surveillance in Turkey.
[59]	n = 73 (three with HHMA — two male); ages: six months, 13 months, and five months.	To investigate whether isolated HHMA can be considered a CFTR-related disorder (CFTR-RD) in childhood, given that some patients show CFTR dysfunction without meeting diagnostic criteria.	Severe dehydration, growth failure, anorexia, vomiting, and oliguria.	F508del/R1070W; F508del/L467F/P5L; and R1066H/P5L.	The patients presented with metabolic alkalosis and salt depletion as a manifestation of CFTR dysfunction. There were no pulmonary symptoms, pancreatic insufficiency, or other typical CF manifestations during the nine-year follow-up. Treatment consisted of oral salt supplementation. Sweat test results were negative or borderline.	Isolated HHMA may represent a clinical entity within CFTR-RD in childhood. As these patients do not meet the diagnostic criteria for CF but have a specific manifestation of CFTR dysfunction, this condition could be recognized as a pediatric form of CFTR-RD.
[32]	n = 68 (12 with HHMA; age range from three to 58 months).	To describe the prevalence and clinical characteristics of HHMA in children with CF.	Hyponatremia, hypokalemia, hypochloremia, metabolic alkalosis, dehydration, and a high rate of malnutrition (91.6% of patients with HHMA).	No specific genetic data were reported for this sample.	Higher prevalence of HHMA (32.4%) in infants, seasonal trend (73.0% of cases occurring in summer), and the need for increased awareness for early diagnosis, especially in hot regions.	HHMA is a common complication in children with CF, especially in infants and in hot climates, with risk of recurrence and morbidity. Early recognition is essential to prevent complications.

Ref	Sample	Objective	Clinical presentation	Genetics	Outcome	Conclusion
[60]	n = 9 (eight male); HHMA occurred during the hottest summer; a total of 57 retrospective episodes in 43 people with CF.	To investigate an outbreak of HHMA in people with CF during a period of extreme heat in Turkey.	Hyponatremic and hypochloremic metabolic alkalosis, with hypokalemia and severe dehydration. Symptoms of tetany, seizures, hypoventilation, reduced cardiac output, and cardiac arrhythmias were reported. A 23-month-old patient died six hours after admission despite intensive treatment.	F508del (heterozygous and homozygous) and G542X.	The outbreak was associated with high environmental temperatures. Most patients were already on oral salt supplementation but still developed HHMA. Older children were more affected than infants, possibly due to greater heat exposure.	HHMA is a common complication in CF and can be worsened by high temperatures. During heat waves, patients should be closely monitored and receive additional electrolyte supplementation to prevent severe dehydration and metabolic complications.
[13]	n = 241 (HHMA in 29 patients: 14 boys, total of 44 episodes); age ranged from one month to 20 years; mean age at first episode: four months.	Describe the clinical and laboratory characteristics and therapeutic strategies for HHMA in people with CF.	Episodes of hyponatremic and hypochloremic dehydration with metabolic alkalosis, frequently associated with vomiting, loss of appetite, and pulmonary exacerbations. Most patients experienced their first HHMA episode at four months of age (range one to 18 months).	The pathogenic variant F508del was the most common, present in 22.4% of patients with HHMA (three homozygous, seven heterozygous).	Patients were treated with intravenous fluid therapy and oral sodium chloride supplementation. Despite supplementation, 12 of the 29 required hospitalization due to recurrent HHMA episodes. The last recorded episode occurred at 48 months of age.	CF should be considered in the differential diagnosis of hyponatremic and hypochloremic metabolic alkalosis in children, even in the absence of typical symptoms, which may develop later. HHMA is common, especially in contexts of low salt intake, hot climates, and associated lung disease, and may be related to genetic variants. Awareness and early recognition are essential to prevent complications such as chronic alkalosis and hypocalcemic seizures.
[55]	n = 17	To investigate the genetic basis of Bartter syndrome cases in an Iranian cohort and assess the presence of	Patients presented with hypokalemic metabolic alkalosis; some exhibited hypercalciuria or hypocalciuria, and one case showed nephrocalcinosis. The clinical	New pathogenic variant in the *CFTR* gene (S158N) associated with the pseudo-Bartter syndrome phenotype in the	Twelve out of 17 patients (70%) presented a deletion of the *CLCNKB* gene, confirming Bartter syndrome. Four patients were reclassified as having conditions	Deletion of the *CLCNKB* gene was the most frequent alteration, being the main cause of Bartter syndrome in this population. However, 25%

Ref	Sample	Objective	Clinical presentation	Genetics	Outcome	Conclusion
		conditions that mimic the disease, such as CF.	spectrum ranged from early manifestations to late presentations resembling Gitelman syndrome.	absence of respiratory symptoms.	that mimic Bartter syndrome (HHMA), including CF, congenital chloride diarrhea (*SLC26A3*), Pendred syndrome (*SLC26A4*), and apparent mineralocorticoid excess (*HSD11B2*). One case remained without a defined genetic diagnosis.	were reclassified as HHMA due to other genetic diseases, including CF. The study highlights the importance of genetic analysis to correctly differentiate these conditions and avoid diagnostic errors that can impact clinical management.
[37]	n = 12 (seven boys).	To characterize common and rare clinical presentations of CF in children in Saudi Arabia and discuss diagnostic and therapeutic challenges.	The patients exhibited growth failure, respiratory infections, and chronic diarrhea. Eight of the 12 children developed HHMA. Other findings included nasal polyposis, peripheral neuropathy due to vitamin E deficiency, meconium ileus, and cholelithiasis. Colonization by *Pseudomonas aeruginosa* occurred in 67.0% of patients, on average at seven months of age.	No specific genetic data were reported for this sample.	There was an average delay of 11 months in diagnosis, even with severe symptoms. One patient died due to growth failure and respiratory infection by *P. aeruginosa*. The remaining 11 patients were alive at follow-up. The study suggests that CF may be more common in Saudi Arabia than previously thought, especially due to the high rate of consanguineous marriages.	CF is not as rare in Saudi Arabia as previously believed. Early recognition is essential to avoid diagnostic delay and clinical deterioration. The study reinforces the need for greater access to sweat chloride testing and to prevent early colonization by *P. aeruginosa*. Salt supplementation is recommended in hot climates to prevent HHMA.
[61]	n = 202 (11 with HHMA; 6.6%).	To characterize the clinical and genetic profile of CF in an Arab population from Jordan.	75.4% presented classic CF symptoms, including growth failure, malabsorption, and respiratory symptoms. 6.6% (11 patients) had HHMA, characterized by severe hyponatremic dehydration, often following mild episodes of diarrhea during the summer.	Twenty-four mutations in the *CFTR* gene were identified, with a low prevalence of F508del (7.4%). Six mutations were described for the first time.	High infant mortality rate (23% of patients died, most within the first year of life). Identification of 24 variants, including six novel ones (296+9A→T, T338M, T760M, 3670delA, 4006delA, and G1244D). The low frequency of F508del suggests genetic variability in the Jordanian population.	CF may be underdiagnosed in Arab populations. HHMA was observed in a significant number of cases, associated with severe dehydration during summer. The diversity of mutations in the *CFTR* gene reflects the population's admixture. High mortality is related to delayed diagnosis and

Ref	Sample	Objective	Clinical presentation	Genetics	Outcome	Conclusion
						limited access to appropriate therapies.
[10]	n = 7 (four male).	To describe cases of HHMA in people with CF and assess their relationship with chronic salt loss, highlighting the importance of differential diagnosis in cases of unexplained growth failure.	Children with CF presenting hyponatremia, hypokalemia, and hypochloremia associated with metabolic alkalosis. Failure to gain weight despite treatment for CF. None had fever or signs of severe dehydration at admission. Some cases had a history of mild respiratory infections or vomiting.	No specific genetic data were reported for this sample.	Electrolyte replacement (sodium and potassium) resulted in rapid recovery of metabolic status and weight gain. Patients remained clinically stable after electrolyte normalization. Findings suggest underdiagnosis of HHMA, especially during hot periods of the year.	HHMA should be considered in the differential diagnosis of children with CF who do not show adequate growth, especially during hot months. Electrolyte replacement is effective in reversing the condition. Chronic salt loss and activation of the renin-angiotensin-aldosterone system are central mechanisms in the pathophysiology of the disorder.
[38]	n = 3.	To describe metabolic alkalosis with electrolyte disturbances (HHMA) as an initial manifestation of CF in infants.	Infants presented with anorexia, weight loss, previous episodes of gastrointestinal and respiratory infections, hypotonia, growth failure, and electrolyte abnormalities (hyponatremia, hypokalemia, and hypochloremia) associated with metabolic alkalosis.	No specific genetic data were reported for this sample.	Patients showed clinical improvement with appropriate electrolyte replacement, with no recurrences after regular salt supplementation.	Metabolic alkalosis with hyponatremia, hypokalemia, and hypochloremia can be an initial manifestation of CF in infants, often associated with growth failure. The mechanism involves electrolyte losses through sweat, especially during hot periods, leading to activation of the renin-angiotensin-aldosterone system. Electrolyte replacement is essential to restore acid-base balance.
[39]	n = 1 (one month old).	Report a rare case of HHMA as the initial manifestation of CF, particularly triggered by hot climate.	An infant with recurrent episodes of severe dehydration, metabolic alkalosis, electrolyte disturbances (hyponatremia and	No specific genetic data were reported for this sample.	The diagnosis of CF was suggested by hypochloremia, low urinary chloride levels, high sweat chloride concentrations, and	HHMA can be an initial manifestation of CF, especially in hot climates due to excessive chloride loss through sweat. CF should be

Ref	Sample	Objective	Clinical presentation	Genetics	Outcome	Conclusion
			hypokalemia), excessive chloride loss through sweat, and collapse of the right upper lobe.		pulmonary collapse. The patient was treated with oral hydration and nebulization, and a follow-up plan was established including sodium supplementation during hot periods, weight gain monitoring, and vaccination (influenza and pneumococcus).	considered in infants with hypochloremic dehydration, metabolic alkalosis, and low urinary chloride levels, even in the absence of a family history.
[11]	n = 110 (18 with HHMA, nine female) / mean age seven years / mean age at first HHMA episode: three months.	To compare the clinical characteristics and progression of CF in patients with and without HHMA, as well as to evaluate the correlation with genetic variants and *Pseudomonas* colonization.	Recurrent episodes of dehydration, hyponatremia, hypokalemia, hypochloremia, and metabolic alkalosis were observed. The episodes occurred mainly during the summer and in infants, with a median age at the first episode of three months. Three patterns of HHMA were identified: single episode (16.6%), recurrent (66.6%), and chronic (16.6%).	Genetic variants were identified in only 30% of people with CF. Some of the variants found include M2789+5G, N1303K, and F508del.	Patients with HHMA had an earlier diagnosis of CF and a higher rate of *Pseudomonas* spp. colonization before the first year of life (44% vs. 12%). The Shwachman-Kulczycki score was lower in patients with recurrent HHMA (69 vs. 85 in others).	The diagnosis of CF based on a clinical picture compatible with HHMA is more specific for CF than other manifestations. CF should be considered in the differential diagnosis of any child with biochemical features suggestive of Bartter syndrome, especially in countries where neonatal screening and CF testing are not widely available, as the disease may remain monosymptomatic for years.
[33]	n = 98 (three with HHMA).	To investigate the relationship between CF and HHMA by analyzing sweat electrolyte levels and their correlation with clinical presentation and metabolic changes.	Children with CF who developed HHMA characterized by metabolic alkalosis, hypokalemia, hypochloremia, and dehydration. Symptoms included respiratory infections, growth failure, anorexia, vomiting, diarrhea, and excessive	No specific genetic data were reported for this sample.	HHMA was more frequent in the early years of life due to excessive electrolyte loss through sweat. Hypokalemia was exacerbated by secondary hyperaldosteronism, identified by elevated potassium levels in sweat in some cases.	HHMA in CF results from chronic sodium and chloride loss through sweat, with a compensatory hyperaldosteronism response that worsens hypokalemia. Measuring potassium in sweat may serve as a risk marker for this condition. Rigorous electrolyte

Ref	Sample	Objective	Clinical presentation	Genetics	Outcome	Conclusion
			electrolyte loss through sweat.			monitoring and adequate supplementation are recommended to prevent metabolic complications.
[8]	n = 1 (male, six months).	Report a rare case of CF presenting initially as HHMA.	Infant with recurrent episodes of diarrhea and vomiting, dehydration, hyponatremia, hypochloremia, mild hypokalemia, and metabolic alkalosis. The patient experienced recurrent hospitalizations over two years without respiratory manifestations, initially suggesting a diagnosis of HHMA.	F508del/F508del.	The definitive diagnosis of CF was established at eight years old, following a severe episode of gastroenteritis with hypovolemic shock and respiratory infection, confirmed by sweat testing and genetic analysis. The patient started standard CF treatment, with clinical and laboratory improvement.	In the presence of hypochloremic and hypokalemic metabolic alkalosis, the possibility of CF should always be considered, even without typical clinical manifestations, to avoid delays in diagnosis and treatment.
[14]	n = 1 (male, nine years).	To discuss the presentation, management, and prevention of seasonal HHMA in older children with CF, highlighting the importance of electrolyte monitoring during periods of extreme heat.	Hypokalemia, hypochloremia, and metabolic alkalosis without renal tubular disease, associated with dehydration episodes exacerbated by gastroenteritis and extreme heat. In both episodes, there was significant worsening of pulmonary function.	F508del/F508del.	The patient recovered after intravenous electrolyte and fluid replacement, along with antibiotic treatment for respiratory exacerbations. Oral salt supplementation alone was insufficient to prevent new episodes of HHMA.	HHMA can occur recurrently in older children, especially during periods of intense heat. Salt supplementation should be combined with electrolyte monitoring and early interventions to prevent severe complications and deterioration of pulmonary function.
[34]	n = 12 (infants diagnosed by newborn screening, five with HHMA – 41.6%).	To describe the prevalence of HHMA and its relationship with the nutritional status of children with CF diagnosed through newborn screening.	HHMA characterized by hyponatremia, hypokalemia, metabolic alkalosis, and dehydration. Mild clinical manifestations included vomiting, cough, and weight loss. No patients presented seizures or tetany.	A homozygous pathogenic variant R1162X was found in one patient and F508del in another. Three patients did not receive a diagnosis.	There was progressive improvement in nutritional status after CF treatment and early correction of the electrolyte disturbance associated with HHMA. Early screening and appropriate management were	The prevalence of HHMA was high among infants with CF in hot climate regions. Early diagnosis and nutritional intervention enabled rapid recovery and prevented serious complications.

Ref	Sample	Objective	Clinical presentation	Genetics	Outcome	Conclusion
					effective in preventing severe complications.	
[40]	n = 1 (male, 4.5 months).	To report a clinical case of HHMA associated with CF, highlighting the diagnosis and clinical management.	The patient presented with a dry cough lasting two weeks unresponsive to antibiotics, anorexia, and weight loss.	No specific genetic data were reported for this sample.	The patient showed clinical improvement with electrolyte replacement, vitamin supplementation, pancreatic enzymes, and respiratory physiotherapy.	In infants with CF who exhibit failure to thrive, HHMA should be considered and investigated for appropriate management and complication prevention.
[52]	n = 1 (male, four years).	To report a rare case of HHMA as the sole manifestation of CF and describe the associated genetic and phenotypic characteristics.	Severe dehydration and lethargy without a history of respiratory or gastrointestinal symptoms.	Heterozygous pathogenic 711+1G>T variant with the IVS8-5T-TG11 allele in the *CFTR* gene; M470C/V470 genotype.	Fluid replacement therapy corrected the biochemical disturbances within two days, with a weight gain of 3 kg.	This is the first reported case of the combination of the 711+1G>T variant with the IVS8-5T-TG11 allele resulting in HHMA as the initial manifestation of CF. The report underscores the need to include CF in the differential diagnosis of children with HHMA, especially in countries without newborn screening.
[41]	n = 1 (male, seven months).	To report a case of HHMA in an infant with CF, highlighting vomiting as an early clinical marker of metabolic decompensation.	Vomiting and lethargy, with dehydration, hyponatremia, hypokalemia, hypochloremia, and metabolic alkalosis.	D110H/F508del.	The patient was treated with intravenous fluids and sodium and potassium supplementation, resulting in electrolyte normalization.	Vomiting may be the only initial sign of metabolic decompensation in HHMA in people with CF, underscoring the importance of early differential diagnosis to prevent complications.
[42]	n = 1 (female, eight months old).	To report a case of HHMA in a patient with CF who had a negative neonatal screening, highlighting heat as a precipitating factor.	Severe dehydration, hypokalemia, and metabolic alkalosis triggered by heat exposure.	G126D/F508del.	Management was carried out with intravenous hydration, resulting in clinical stabilization.	HHMA should be considered a possible complication in people with CF, even when neonatal screening is negative, especially in situations of heat exposure.

Ref	Sample	Objective	Clinical presentation	Genetics	Outcome	Conclusion
[12]	n = 5 (three females) / age between three and 17 months.	To describe the clinical presentation of HHMA in various initial manifestations of CF, emphasizing the differential diagnosis with HHMA.	Difficulty gaining weight; recurrent respiratory infections; meconium ileus progressing to ileostomy; asymptomatic cases identified through screening.	Described mutations: 621+1G>T/2789+5 G>A, F508del/F508del, and F508del/G542X.	Management ranged from nutritional support and correction of electrolyte imbalances to respiratory support, resulting in progressive clinical improvement.	HHMA should be considered in the differential diagnosis of children with CF, especially in the presence of poor weight gain and gastrointestinal or respiratory manifestations.
[43]	n = 1 (female, three months).	To report a case of HHMA as the initial presentation of CF in an infant, highlighting the persistence of metabolic alkalosis even after hospital discharge.	Cough, soft stools, nonbilious vomiting, abdominal distension, and difficulty gaining weight.	No specific genetic data were reported for this sample.	The treatment included intravenous fluid therapy with saline and dextrose, antibiotics, pancreatic enzymes, vitamins, nutritional supplements, MCT oil (Medium-Chain Triglycerides), and bronchodilators; the patient was discharged after 10 days, still exhibiting metabolic alkalosis.	The case highlights the importance of monitoring persistent metabolic disturbances in people with CF, even after clinical improvement of respiratory and gastrointestinal symptoms. Nutritional and electrolyte follow-up is essential.
[44]	n = 1 (female, six months).	To report a severe complication of HHMA in an infant with CF, emphasizing the need to monitor salt supplementation in breastfed infants.	Neonatal screening negative for CF. At six months, presented persistent metabolic alkalosis, chronic dehydration, and poor nutritional status, being exclusively breastfed.	F508del/711+1G>T	Treatment included correction of electrolyte disturbances and rehydration. After discharge, metabolic acidosis was managed with sodium bicarbonate supplementation. Nutritional status improved.	This case underscores the importance of early diagnosis, close monitoring of hydration, and guidance on salt supplementation in breastfed infants with CF to prevent severe complications of HHMA.
[9]	n = 12 (five males; mean ages (± SD): onset of HHMA 4.0 (± 1.7) months and CF diagnosis 28.6 (± 37.5) months).	To characterize the clinical presentations, genetics, and management of HHMA associated with CF in Chinese children.	Recurrent/persistent pneumonia (91.7%), pancreatitis (83.3%), vomiting/diarrhea (66.7%), growth failure (58.3%), and liver disease (58.3%). Infection with *P. aeruginosa* (83.3%) and *Staphylococcus aureus* (75.0%).	Nineteen variants in the *CFTR* gene, including 10 novel ones. The most common variant was G970D (16.6% allele frequency). F508del was observed for the first time in	Electrolyte and acid-base abnormalities resolved after resuscitation with 3% hypotonic saline or normal saline.	HHMA may be an underdiagnosed initial manifestation of CF in China. Early recognition is essential due to the high frequency of associated complications. The G970D variant shows a distinct ethnic pattern

Ref	Sample	Objective	Clinical presentation	Genetics	Outcome	Conclusion
				homozygosity in a Chinese patient.		compared to Caucasian populations.
[45]	n = 1 (nine months old).	Discuss the differential diagnosis and management of metabolic alkalosis in children, highlighting the importance of investigating CF as a cause of HHMA.	Severe dehydration, hyponatremia, hypokalemia, hypochloremia, and metabolic alkalosis, without a history of vomiting or diarrhea.	F508del/Asp110His.	Diagnosis confirmed of HHMA secondary to CF by sweat test and genetic analysis.	CF should be considered in the differential diagnosis of children with hypochloremic and hypokalemic metabolic alkalosis without an apparent gastrointestinal cause. The sweat test remains essential.
[46]	n = 1 (75 days old).	Report of a rare case of CF presenting initially as HHMA, with early pulmonary colonization and an uncommon genotype.	Failure to gain weight, severe dehydration, hyponatremia, hypokalemia, metabolic alkalosis, and pulmonary infection with primary colonization by *P. aeruginosa*.	3849+1G>A/4382delA.	Progressive correction of hyponatremia and hypokalemia with intravenous replacement and oral sodium supplementation, with improvement in weight gain.	The identified genotype is rare and associated with electrolyte loss and pulmonary involvement. The importance of detailed genetic screening in atypical cases of HHMA is highlighted.
[47]	n = 1 (female, six months old).	To report a case of HHMA associated with CF in a Chinese child, discussing its rarity in Asia and the challenges for early diagnosis.	Recurrent pneumonias, persistent cough, wheezing for three months of age, hypokalemia, metabolic alkalosis, moderate dehydration, growth failure, and mild bilateral bronchiectasis.	G178R/G178R.	Diagnosis confirmed by genetic analysis. Improvement after antibiotics, respiratory support, hydration, and nutritional supplementation.	HHMA can be the first manifestation of CF, including in Asian populations. Early diagnosis requires clinical awareness, especially where newborn screening is not widely implemented.
[48]	n = 1 (female, four months old).	To report a case of CF presenting a clinical picture similar to HHMA in an infant, highlighting the value of repeated sweat testing.	Dehydration, hyponatremia, hypokalemia, hypochloremia, and metabolic alkalosis. Hyperaldosteronism and hyperreninemia, without vomiting or alkali ingestion. Initial suspicion of Bartter syndrome. Sweat sodium	No specific genetic data were reported for this sample.	Initial diagnosis undefined; at three years old, the patient was asymptomatic, without electrolyte abnormalities, but with persistently elevated sweat tests, confirming CF.	CF can manifest as HHMA in infants with excessive salt loss through sweat. Hypovolemia leads to secondary hyperaldosteronism. The sweat test should be repeated in suspected Bartter cases to avoid misdiagnosis.

Ref	Sample	Objective	Clinical presentation	Genetics	Outcome	Conclusion
			concentration initially normal, then elevated.			
[49]	n = 1 (male, five months old).	To demonstrate that a normal sweat test does not exclude CF and to highlight the importance of genotyping in suspected cases.	Fever and cough, initial diagnosis of broncho-pneumonia and pseudo-Bartter. Weight loss over the past 1.5 months. Hyponatremia, hypochloremia, metabolic alkalosis. Normal sweat tests.	E92K/F1052V.	Final diagnosis of CF based on clinical presentation and genetic analysis, despite a negative sweat test.	Although the sweat test is the gold standard for CF, false-negative results can occur. Genetic analysis is essential when there is strong clinical suspicion, reinforcing the importance of combining diagnostic methods.
[50]	n = 3 (two males) / diagnoses at four, seven, and six months.	To report three cases of infants with late diagnosis of CF, with HHMA as the initial manifestation, associated with the K162E variant in the *CFTR* gene.	Severe hyponatremic-hypochloremic dehydration (HHMA). Chronic cough. Inadequate weight gain. Respiratory infections, including *P. aeruginosa* in one case. Preserved pancreatic function.	The K162E variant was found in association with other variants (2307insA, 541delC, and R334W).	All patients were diagnosed in childhood following severe dehydration. They are currently stable with salt supplementation and respiratory physiotherapy.	The K162E variant is potentially pathogenic, possibly triggering HHMA as the first manifestation of CF. The study emphasizes the importance of reviewing neonatal screening criteria to detect fewer common variants in diverse populations.
[54]	n = 1 (female, 21 years old).	To report a case of Bartter-like metabolic alkalosis induced by aminoglycoside (tobramycin) in a patient with CF.	Patient with CF, diabetes mellitus, and allergic bronchopulmonary aspergillosis. Hospitalized with dyspnea and hemoptysis. Developed respiratory acidosis and, on the 7th day, presented hypokalemia, hypomagnesemia, hypocalcemia, and metabolic alkalosis lasting two weeks despite aggressive replacement therapy.	No specific genetic data were reported for this sample.	Treated with discontinuation of tobramycin, spironolactone, and amiloride. Electrolytes normalized by day 25, except magnesium. Discharged with magnesium supplementation and amiloride.	The Bartter-like condition was induced by tobramycin, through activation of the calcium-sensing receptor causing tubular dysfunction. This is the first documented association of this effect in a patient with CF.
[53]	n = 1 (male, nine years old).	To report a case of HHMA as an atypical initial	Child presenting with vomiting, abdominal pain, and mild to	No specific genetic data were	Fluid and electrolyte replacement corrected the metabolic disturbances.	HHMA can be the first manifestation of CF in children without prior

Ref	Sample	Objective	Clinical presentation	Genetics	Outcome	Conclusion
		manifestation of CF.	moderate fever. Laboratory tests revealed metabolic alkalosis, hypokalemia, hyponatremia, and hypochloremia. Initial suspicion was dehydration. Sweat test performed after clinical evaluation confirmed CF.	reported for this sample.	CF diagnosis was confirmed, and appropriate follow-up was initiated.	diagnosis, especially in regions without universal newborn screening. Metabolic alkalosis with electrolyte disturbances should prompt investigation of CF
[51]	n = 1 (male, 15 years old).	To report an atypical case of CF manifested in adolescence as HHMA associated with obesity and hypertension.	Recurrent episodes of malaise associated with dehydration and acute renal failure. Tests showed hypochloremic metabolic alkalosis, hyponatremia, and hypokalemia. The patient was obese, hypertensive, and had a history of mild respiratory symptoms.	F508del/G126D.	Definitive diagnosis of CF was established late due to the unusual clinical presentation. Specific treatment was initiated after correction of the electrolyte disturbances.	This is the first reported case of CF presenting as HHMA in an obese and hypertensive adolescent. The report underscores the importance of considering CF as a differential diagnosis in cases of hypochloremic metabolic alkalosis, even in adolescents and with atypical phenotypes.
[56]	n = 1 (male, nine months old).	To report the use of modified oral rehydration therapy as an alternative to intravenous hydration in a patient with CF and HHMA, in the context of difficult vascular access.	Pneumonia with progression to diarrhea, moderate dehydration, hyponatremia, and metabolic alkalosis.	No specific genetic data were reported for this sample.	The modified oral rehydration therapy (with higher NaCl concentration and no bicarbonate) was effective in stabilizing electrolytes and fluids, controlling diarrhea, and allowing subsequent successful intravenous hydration.	Modified oral rehydration may be a safe and effective alternative for managing HHMA in CF when intravenous access is not feasible, highlighting the need for further research to validate optimized protocols.
[29]	n = 24 (11 with HHMA) / 15 males / mean age 5.3 ± 4 years / symptom onset 3.3 ± 3 months.	To analyze the genotype and phenotype of children with CF diagnosed in early childhood and to evaluate the correlation	The main complaints at admission were diarrhea (79.2%), respiratory manifestations (45.8%), and growth retardation (50.0%). Eight cases (33.3%) presented with dehydration,	Eighteen cases with positive *CFTR* results: F508del was present in nine cases (50.0%), with four being homozygous; in two	The patients received nutritional support, electrolyte and fluid replacement in cases of diarrhea and HHMA, pancreatic enzymes for exocrine insufficiency, and antibiotic	The frequency of the F508del variant was higher than reported in other cohorts in Turkey. The study emphasizes the importance of early diagnosis and proper management of HHMA

Ref	Sample	Objective	Clinical presentation	Genetics	Outcome	Conclusion
		between pathogenic variants in the *CFTR* gene and clinical manifestations, focusing on HHMA.	frequently accompanied by diarrhea (87.5%). Excessive sweating was reported in five patients, and seizures occurred in two. Among the 11 cases with HHMA, diarrhea was the predominant complaint in eight (72.7%). Hospitalization rates were higher in patients with HHMA (72.7% vs. 53.8%).	severe cases, two variants were identified (details not specified); one case was lost to follow-up; six patients had no variant identified.	therapy/mucolytics for respiratory symptoms. A higher rate of severe growth retardation was observed in children diagnosed before one year of age (62.5%). Among the HHMA cases, 10 (62.5%) were diagnosed within the first year of life, while only 1 (12.5%) occurred in children diagnosed after that age.	to reduce nutritional and respiratory complications in children with CF.

±, plus or minus, %: percentage, *CFTR*: Cystic Fibrosis Transmembrane Conductance Regulator, CLCNKB: Chloride Voltage-Gated Channel Kb, HSD11B2: Hydroxysteroid 11-Beta Dehydrogenase 2, Kg: kilogram, N: number of individuals, Na: sodium, NaCl: sodium chloride, SD: standard deviation, SLC26A3: Solute Carrier Family 26 Member 3, SLC26A4: Solute Carrier Family 26 Member 4.
